# Coloring Outside the Lines: Exploiting Pigment–Protein Synergy for Far-Red Absorption in Plant Light-Harvesting Complexes

**DOI:** 10.1021/jacs.3c13373

**Published:** 2024-01-29

**Authors:** Eduard Elias, Katrin Brache, Judith Schäfers, Roberta Croce

**Affiliations:** Department of Physics and Astronomy and Institute for Lasers, Life and Biophotonics, Faculty of Sciences, Vrije Universiteit Amsterdam, de Boelelaan 1081, 1081 HV Amsterdam, The Netherlands

## Abstract

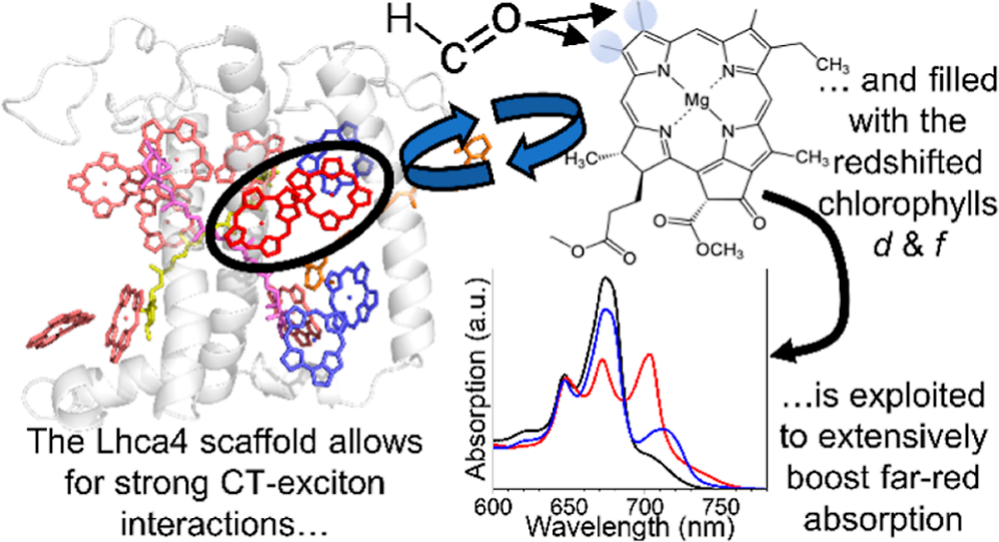

Plants are designed to utilize visible light for photosynthesis. Expanding this light absorption toward the far-red could boost growth in low-light conditions and potentially increase crop productivity in dense canopies. A promising strategy is broadening the absorption of antenna complexes to the far-red. In this study, we investigated the capacity of the photosystem I antenna protein Lhca4 to incorporate far-red absorbing chlorophylls *d* and *f* and optimize their spectra. We demonstrate that these pigments can successfully bind to Lhca4, with the protein environment further red-shifting the chlorophyll *d* absorption, markedly extending the absorption range of this complex above 750 nm. Notably, chlorophyll *d* substitutes the canonical chlorophyll *a* red-forms, resulting in the most red-shifted emission observed in a plant light-harvesting complex. Using ultrafast spectroscopy, we show that the introduction of these novel chlorophylls does not interfere with the excited state decay or the energy equilibration processes within the complex. The results demonstrate the feasibility of engineering plant antennae to absorb deeper into the far-red region while preserving their functional and structural integrity, paving the way for innovative strategies to enhance photosynthesis.

## 1 Introduction

Light harvesting is the first step of photosynthesis, the process by which plants, algae, and some bacteria convert light energy to chemical energy. The capture of light takes place in the thylakoid membrane of the chloroplasts leading to the direct conversion of light energy into chemical energy in the form of ATP and NADPH. This process is mediated by two major multiprotein complexes, photosystem I (PSI) and photosystem II (PSII), consisting of an outer antenna system composed of several light-harvesting complexes (LHCs) that surround a core hosting the photochemical reaction center (RC). The primary role of the antennae is to absorb light and transfer excitation energy to the RC where it is used to promote charge separation.^1^

The LHCs of plants are all members of the same nuclear-encoded protein superfamily and display a high degree of sequence and structural homology.^2^ The LHC proteins consist of three membrane-spanning α-helices and two amphipathic helices exposed to the thylakoid lumen. The LHCs of plants bind 11–16 chlorophylls (Chls), and 3–4 carotenoids (Cars).^2^ The apoproteins act as scaffolds that arrange the pigments, controlling the interpigment distances and mutual orientations. The energies of the pigments are tuned by their environment and the excitonic interactions between them. In this way ultrafast, efficient and directional excitation energy transfer is ensured.^1^

**Figure 1:**
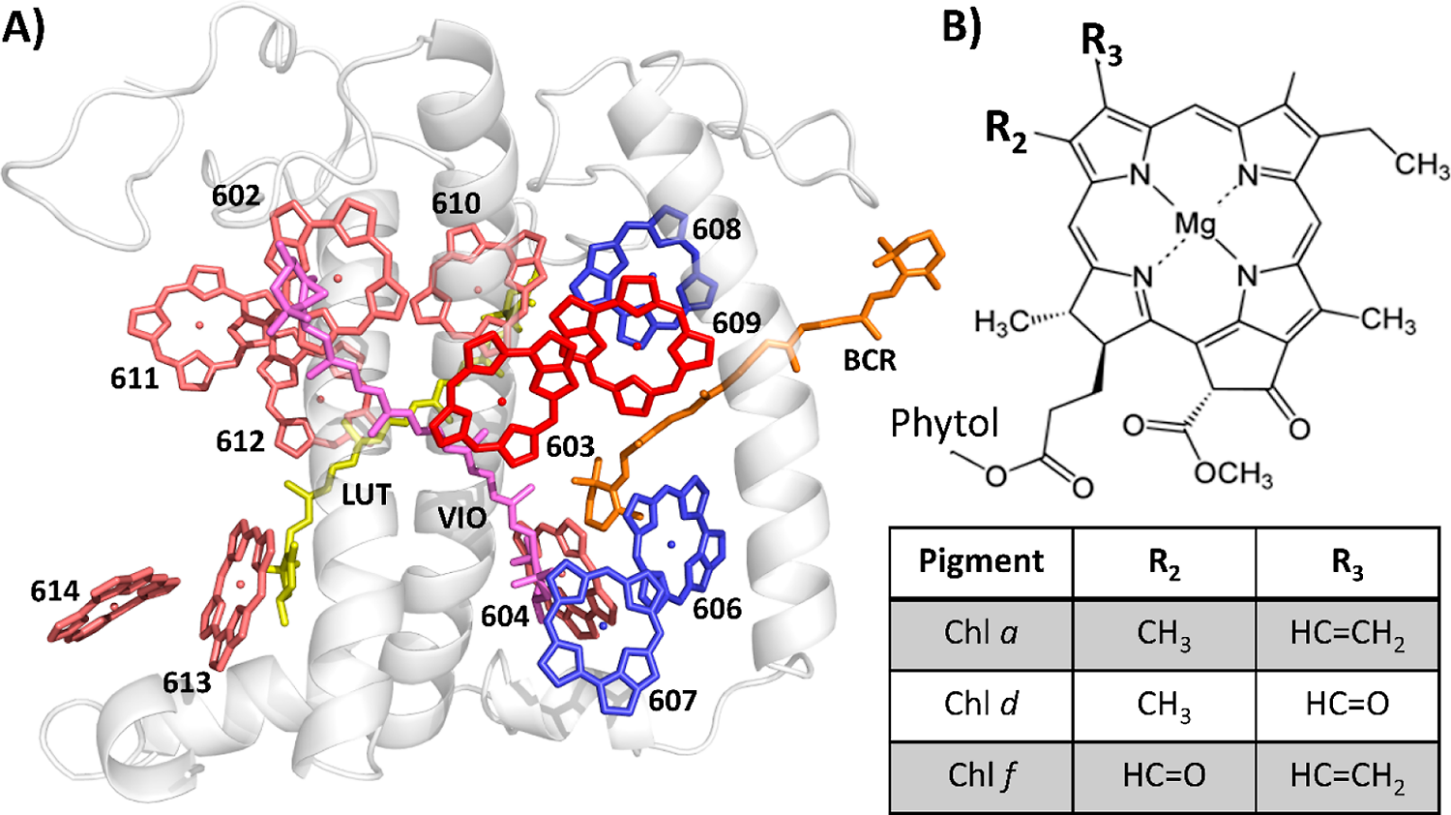
Lhca4 and Chl structures. (A) Crystal structure of plant Lhca4 of *Pisum sativum* (PDB: 5L8R),^25^ showing the protein in gray, Chls *a* 603 and 609 in rose, all other Chls *a* in red, Chls *b* in blue, Violaxanthin in magenta, Lutein in yellow, and β-carotene in orange. (B) Chl molecular structure. The table indicates the chemical groups that are present for Chls *a*–*f* at the R_2_ and R_3_ position.

The tuning of the pigment energies is especially well achieved in the PSI-specific antenna complex Lhca4. Lhca4 has the red-most absorption and emission spectra of all plant LHCs, with Chl *a* spectral forms showing maxima up to 710 nm, which is red-shifted by about 30 nm with respect to the red-most forms of Lhcbs. The Lhca4 structure is shown in Figure 1A. The origin of its red-shifted absorption could be attributed to the Chl *a* 603–Chl *a* 609 dimer^3,4^ and to mixing between an exciton and a charge-transfer (CT) state.^5−8^ Apart from a significant red-shift, the main absorption and emission bands of these Chls are characterized by a substantially broadened profile and a large Stokes shift.^8^

Despite the extensive tuning of the pigment energies in the LHCs, the light that can be absorbed and efficiently used for photosynthesis in plants is mainly limited to the visible range and is constrained by the inherent absorption properties of the red-most absorbing photosynthetic pigment in plants, i.e., Chl *a*. This represents a limitation, especially for leaves in the shade, which is a common situation in the field where densely packed crops that compete for light cast shadows on their neighboring plants and their own lower leaves. Filtered light at the bottom of a canopy is however still rich in far-red photons (FR, λ = 700–800 nm).^9^ It is also a limitation for the use of LHCs as natural light-harvesting materials in nanoscale photonic applications, where the interception of a larger spectral range is desired.

While plants are not able to extend their absorption spectrum further into the far-red, several cyanobacteria have developed FaRLiP (far-red light photoacclimation) responses that permit them to survive in shaded environments. In far-red light these cyanobacteria synthesize the far-red absorbing Chls *d* and *f* and incorporate them into newly synthesized proteins.^10−12^ To engineer a similar response in plants might be a promising avenue for increasing crop yields.^1,9,13,14^

Chls *d* and *f* are chemically very similar to the plant native Chls *a* and *b*: just as Chl *b*, Chls *d* and *f* only differ from Chl *a* by a formyl group that substitutes a vinyl or a methyl in position 3 and 2 (IUPAC numbering, see Figure 1B), respectively. Furthermore, Chl *d* was shown to be able to bind to the major LHC of plants, LHCII, and the resulting complex showed a significant increase in far-red absorption while its functional properties were intact.^15^

While LHCII is predominantly associated with PSII, the addition of far-red absorbing Chls to the antenna of PSI might be an even more straightforward avenue for increasing functional light absorption in the shade. PSI already contains several long-wavelength Chl *a* forms (called red forms), but the excitation energy in PSI is nevertheless used for photochemistry with a quantum efficiency that approaches unity.^16,17^ Strikingly, in FaRLiP PSI, efficient trapping was observed from a Chl *f*-cluster emitting at 790 nm,^18^ while the reaction center in these systems was shown to be very similar to its canonical counterpart.^19,20^ More recently a cyanobacteria strain grown in low light was shown to only expand the absorption of PSI to the far-red expressing and assembling a new type of antenna.^21,22^ Moreover, the location of the red forms in PSI have been shown to be of minor importance for the trapping time, what counts most are the number and energy of low energy forms.^23,24^ These data together strongly suggest that the incorporation of Chls *d* and *f* into the antenna of plant PSI should not significantly hamper its photosynthetic efficiency and that this could be an effective tool for boosting the absorption of photons in low light environments.

In this work, we aimed to synergetically combine the strategies used by plants and FaRLiP cyanobacteria to shift the absorption to the red by introducing the red-shifted Chls *d* and *f* into the red-shift-enabling protein Lhca4. We did so by reconstituting the Lhca4 apoprotein with pigment mixes containing Chl *d* or *f*. To probe their competitiveness in binding with respect to native plant pigments, Chls *a* and *b* were also included in the mixes. The same procedures were followed for the Lhca4-N47H mutant. N47 is the axial ligand of Chl *a* 603 and its mutation to H was shown to abolish the far-red absorption, without the loss of Chl *a* 603.^3^ Comparison of Lhca4-WT to the N47H mutant allows for a more detailed investigation of the consequences of the binding of Chl *d* and *f* on the red-form. The functional properties of the complexes were investigated by using a variety of biochemical and spectroscopic techniques.

## 2 Experimental Section

### 2.1 In Vitro Reconstitutions

The N47H mutant of Lhca4 was obtained by modifying the pET-28a (+) vector that contained the coding sequence of Lhca4 of*Arabidopsis thaliana*.^26^ The apoproteins of Lhca4 and the N47H mutant were overexpressed in *Escherichia coli* [Rosetta 2(DE3)] and purified as inclusion bodies. *In vitro* reconstitution experiments were performed as reported in ref (27). Chls *a*, *b,* and Cars were extracted from spinach leaves.^27^ Chl *d* was extracted from *Acaryochloris marina* cells as described in ref (28). Chl *f* was extracted from far-red light grown *Chroococcidiopsis thermalis* cells as described in ref (29). For the reconstitutions, 800 μg of inclusion body, 160 μg of Cars, and 500 μg of Chls were used. For the Chl *a*/*b* reconstitutions, the Chl *a*/*b* ratio in the pigment mix was 3.0, as this yields recombinant Lhca4 complexes with similar properties as its native counterpart (see, e.g., ref (30)). For the reconstitutions with 3 different Chl types (Chl *a*/*b*/*d* and Chl *a*/*b*/*f*) the pigment mix contained equal amounts of each Chl type to probe on an equal footing the competitiveness of binding of the three Chls to Lhca4. The reconstituted complexes were purified by His-tag Ni-affinity chromatography followed by sucrose density gradient ultracentrifugation with a 0.1–1.0 M sucrose gradient containing 0.06% *n*-dodecyl-β-D-matloside and 10 mM Hepes at pH 7.5, centrifuging at 41,000 rpm (Beckman Coulter, SW41 rotor) at 4 °C for 17 h.^27^ The absorption and emission spectra of the different replicas were very similar, although there was some variation (see Figures S1 & S2). This variation is addressed in the Supporting Information Text S1.

### 2.2 Steady-State Spectroscopy

Absorption spectra were recorded on a Varian Cary 4000 UV–vis spectrophotometer. For measurements at 77 K, a home-built liquid-nitrogen-cooled device was used. The samples were supplemented with 70% (v/v) of glycerol to prevent the formation of ice crystals. Emission spectra were recorded on a HORIBA JobinYvon-Spex Fluorolog 3.22 spectrofluorimeter at an optical density of <0.05 cm^–1^ at the Q_*y*_ maximum. Circular dichroism (CD) spectra were measured with a Chirascan CD spectrophotometer at 10 °C.

### 2.3 Pigment Composition Analysis

The pigments were extracted from the LHCs with 80% acetone. The Chl *a*/*b*, *a*/*b*/*d*, *a*/*b*/*f* , and Chl/Car ratios were estimated by fitting the 80% acetone absorption spectrum with the spectra of the individual pigments in the same solvent.^29,31^ The relative Car ratios were further analyzed using HPLC. The details of this combined approach can be found in ref (32).

### 2.4 Time-Resolved Fluorescence

Time-resolved fluorescence measurements were performed using a time-correlated single-photon counting (TCSPC) setup (PicoQuant FluoTime 200) at 10 °C. The concentration of the samples was <0.05 cm^–1^ at the Q_*y*_ maximum. A laser diode provided the pulsed excitation light at a frequency of 10 MHz and a center wavelength of 466 nm. The instrument response function was determined to be 92 ps (fwhm) measuring the fluorescence decay of a pinacyanol iodide dye dissolved in methanol that has a lifetime of 6 ps.^33^ Power studies were performed to exclude annihilation effects from the measurements.

### 2.5 Time-Resolved Absorption

Transient absorption measurements were performed using a home-built setup previously described.^15^ A coherent MIRA mode-locked Ti/Sa oscillator in combination with a Coherent Rega 9050 regenerative amplifier provided ∼70 fs pulses, centered around 800 nm at a repetition rate of 40 kHz. These pulses were directed in 8:2 ratio to the pump path and probe path, respectively. The pump pulse was tuned to 642 nm using a Coherent OPA 9400 optical parametric amplifier. The spectral bandwidth of the pulse was restricted to 10 nm using an interference filter. The probe pulse was created by means of white-light supercontinuum generation by focusing the 800-nm output of the regenerative amplifier on a YAG crystal. The time delay between the pump and the probe pulse was varied up to 3.5 ns using a retroreflector mounted on a motorized translation stage. Optical chopping of the pump and probe pulses was possible on a shot-to-shot basis using two AA OPTO-ELECTRONIC acousto-optic modulators controlled by a Stanford Research Systems dg645 digital delay generator that was synced to the regenerative amplifier frequency. This allowed to actively correct for dark current and scattering. The spectra of the probe pulses were recorded using a Chromex 250IS spectrograph and a Entwicklungsbüro EB Stresing CCD camera. The polarization between the pump and the probe pulse was set at the magic angle (54.7°) using a Berek’s variable waveplate and a polarizer. The samples were measured at OD < 0.6 mm^–1^ in a 1-mm cuvette and constantly shaken throughout the measurements. Power studies were performed to exclude the presence of annihilation dynamics in the measurements (see Figure S12). Additionally, the excitation density [estimated using *N*_Chl_ × 1/2 × ΔOD_max_/OD_max_,^34^ with *N*_chl_ being the number of Chls per monomer (12)] is below 6% for every measurement, suggesting that the portion of annihilation in the measurements is negligible. The sample integrity (stability) after the measurement was checked by confirming that the emission spectra before and after the measurement were identical.

### 2.6 Global and Target Analysis of Time-Resolved Measurements

To describe the spectral–temporal evolution in the time-resolved data sets (ψ), global analysis was performed. For the time-resolved fluorescence measurements, which were recorded with the TCSPC setup, global analysis using a parallel scheme yields decay-associated spectra (DAS) and associated time constants τ, which describe the data according to the following formula
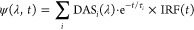


where IRF(*t*) is the instrument response function. The same analysis applied to the time-resolved absorption measurements, which were measured with a pump–probe setup, produced decay-associated difference spectra (DADS).^35^ Global analyses of the time-resolved fluorescence measurements were performed using the TRFA Data Processor Advanced Software^36^ and the measured IRF (see the Time-Resolved Fluorescence section). Global analyses of the time-resolved absorption data were performed with the pyglotaran Python package.^37−39^ For the time-resolved absorption measurements, the IRF was not measured but modeled as a Gaussian with fitted fwhm in the range from 87 to 125 fs.

For the samples reconstituted with Chl *f* (Lhca4-abf and N47H-abf) a target analysis was performed on the transient absorption data to separate the kinetics of the complexes that do contain Chl *f* and those that do not. The target models consisted of two independent sequential schemes: one sequential scheme represents the complexes without Chl *f* and the other the complexes with Chl *f*. The sequential scheme for the complexes without Chl *f* (those containing only Chl *a* and *b*) was constrained to have the same kinetic rates and species-associated difference spectra (SADS) as the evolution-associated difference spectra (EADS) that arose from the global analysis on the respective complexes containing just Chl *a* and *b* (Lhca4-ab and N47H-ab). The sequential scheme for the complexes with Chl *f* contained four compartments. The data sets were fitted until 100 ps, as the analysis aimed at elucidating the excitation energy transfer kinetics rather than the decay, which is best resolved by the TCSPC measurements. As a result of the shorter time window, one decay component was sufficient for this sequential scheme. The area of the first SADS of each independent scheme, which respresents its respective time-zero spectrum, was forced to be equal for both sequential schemes, which allowed for the estimation of the relative portion of complexes with and without Chl *f* in the ensembles as a free fitting parameter. A schematic overview of the kinetic schemes of the target analyses is shown in Figure S13. Target analyses were performed with the pyglotaran Python package.^37−39^

### 2.7 Density Functional Theory Calculations

Density functional theory (DFT) calculations were performed using the ADF2021.101 software^40^ using a B3LYP-D3BJ/TZ2P level of theory.^41−43^ For the calculations the coordinates of Chl *a* 603 and the amino acid F215 were extracted from the crystal structure of Lhca4 of *Pisum sativum* (PDB: 5L8R).^25^ The Chl phytol tail was truncated at the 17^1^ position (IUPAC numbering) and phenylalanine was truncated after the C^β^ position. Chl *a* 603 was converted to Chl *d* by transforming the 3-vinyl group into a formyl group using the build structure module of the CHIMERA software.^44^ Hydrogens were added using the AddHs module of the CHIMERA software.^44^ To calculate the energy of the hydrogen bond between F215 and the Chl *d* 603 3-formyl group, we calculated the energy of Chl *d* 603 (*E*(CLD603)), F215 (*E*(F215)) and of these two interacting molecules together, *E*(CLD603&F215). Energy was evaluated at its energy-minimized geometry. During energy minimizations the central magnesium of the Chl and the C^β^ and C^γ^ atoms of the phenylalanine were position-constrained to conserve the respective orientations of the molecules as resolved in the crystal structure.^25^ The hydrogen bond strength, *E*(hbond), was then calculated as
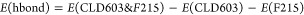


## 3 Results

### 3.1 Chl *d* and *f* Bind to Lhca4 and Significantly Increase the Absorption in the Far-Red

Lhca4 and the Lhca4-N47H mutant were successfully reconstituted with Chls *a* and *b*, Chls *a*, *b,* and *d,* and Chls *a*, *b,* and *f* and in the following these samples are indicated as Lhca4-ab and N47H-ab, Lhca4-abd and N47-abd, and Lhca4-abf and N47H-abf, respectively.

**Figure 2:**
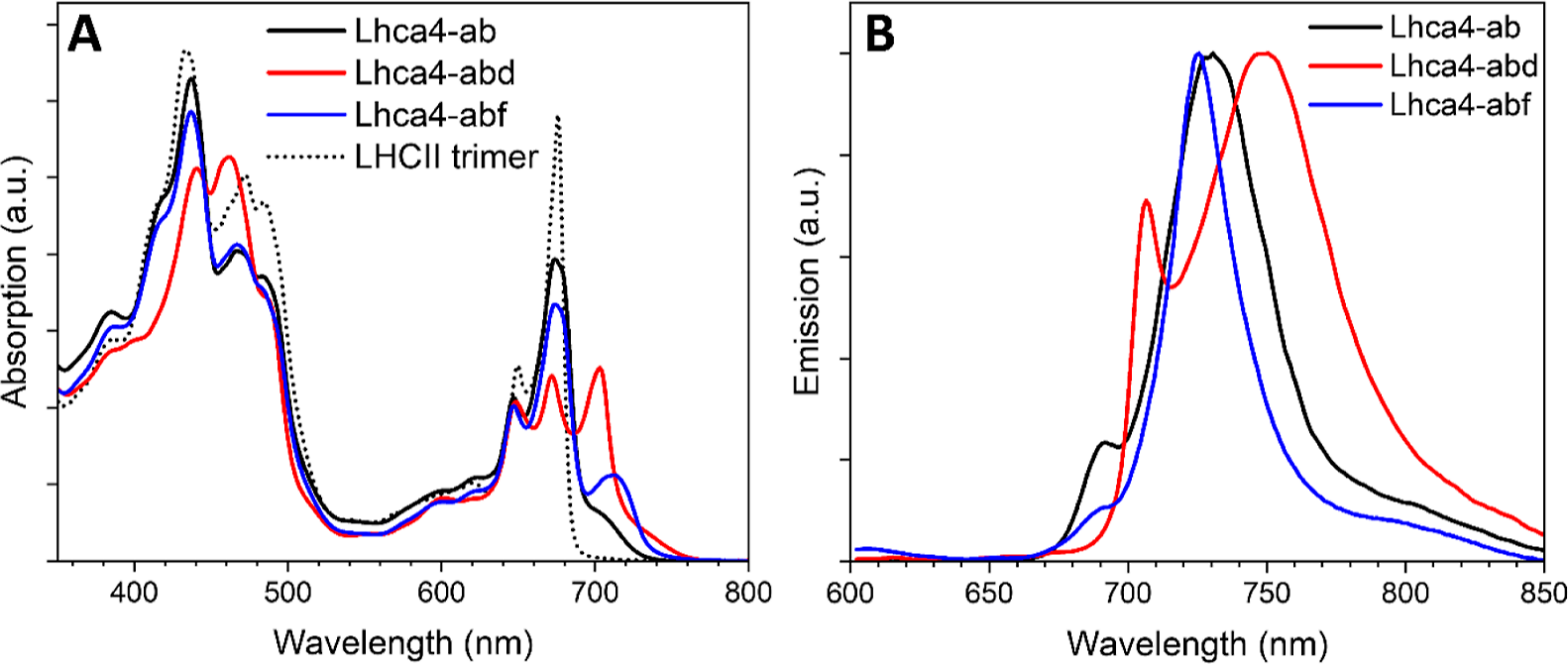
77 K absorption and emission spectra of the Lhca4 complexes. Comparison of absorption (A) and emission spectra (B) of the Lhca4 complexes. The absorption spectra are normalized to their area in the Q_*y*_ region (λ = 630–800 nm) scaled to the Chl content of the complexes as retrieved by the pigment composition analyses (see Table 1). The relative oscillator strengths of Chl *a*:*b*:*d*:*f* were determined to be 1.0:0.67:1.20:1.34 based on their relative absorption in the Q_*y*_ region in 80% acetone.^29,31^ For comparison, the 77 K absorption spectrum of LHCII trimer is also displayed. The emission spectra were measured upon excitation at 500 nm and are normalized by their maxima.

The 77 K absorption spectra of Lhca4-ab (WT), Lhca4-abd and Lhca4-abf are shown in Figure 2A. The most striking feature of (WT) Lhca4 in comparison to Lhcb complexes (see, e.g., LHCII in Figure 2A) is the presence of the red-forms, which in the 77 K absorption spectrum are responsible for the shoulder around 705 nm. Compared to Lhca4-ab, Lhca4-abd and Lhca4-abf show enhanced absorption in the FR region. This demonstrates that Chls *d* and *f* are incorporated into Lhca4. However, the absorption spectrum of Lhca4-abd extends more to the FR than that of Lhca4-abf, which is unexpected because the lowest energy transition of Chl *f* in solution is more red-shifted than that of Chl *d*.^29^

The lowest energy states in the complexes can be better characterized by using low-temperature emission spectra (Figure 2B). The Lhca4-ab emission shows its two typical bands: a small narrow band peaking at 690 nm and a broad band peaking at 730 nm.^3^ The two bands are linked to different conformations of the Lhca4 complex: the broad, red-shifted emission is associated with a conformation characterized by a strong mixing of the CT state with the exciton state.^7,8,45^ Conversely, in the conformation associated with the blue peak, these interactions are absent.^26^ The Lhca4-abd 77 K emission spectrum shows comparable features: the main band has a maximum (∼750 nm) significantly red-shifted and is broader compared to the higher-energy band, which peaks around 705 nm. This shows that Chl *d* in Lhca4-abd is involved in the red-forms, which are considerably lower in energy than in Lhca4-ab. The Lhca4-abf low-temperature emission spectrum has one main band, with a maximum at 725 nm. Strikingly, the band is narrower and peaks at higher energy than the main emission band of Lhca4-ab.

### 3.2 Chl *d* and *f* Compete with Chl *a*

The absorption in the Q_*y*_ Chl *b* region (peak around 650 nm) is similar in all Lhca4 complexes (Figure 2A), which suggests that Chls *d* and *f* do not compete for binding with Chls *b* but rather with Chls *a*. To confirm these observations, we analyzed the pigment composition of the complexes and the results are shown in Table 1. The Lhca4-ab complex has a Chl *a*/*b* ratio of 2.0 and a Chl/Car ratio of 4.9, which is consistent with previous studies on recombinant Lhca4 in which similar pigment mixes were used.^26,30^ The Chl (*a* + *d*)/*b* and the Chl (*a* + *f*)/*b* ratio in Lhca4-abd and Lhca4-abf are 1.7 and 1.8, respectively, indicating that indeed Chls *d* and *f* do not compete in binding with Chl *b*. Upon normalization to 12 Chls per complex, which is the expected number of Chls bound to monomeric Lhca4,^46^ all complexes bind ∼4 Chls *b*. Out of the 8 sites binding Chl *a* in Lhca4-ab, in Lhca4-abd ∼4 are occupied by Chl *d* and 4 by Chl *a*. In Lhca4-abf instead, most sites still bind Chl *a,* and there are only 1.3 Chl *f* per complex. The Chl composition of the N47H-ab and N47H-abf mutants reconstituted with the same pigment mix is largely similar to that of their WTs. N47H-abd instead binds ∼1.8 Chls *a* more than Lhca4-abd, at the expense of Chls *d*, indicating that this mutation influences the affinity of two binding sites.

**Table 1:** Pigment Composition of Lhca4 Complexes^a^

	Lhca4-ab	Lhca4-abd	Lhca4-abf	N47H-ab	N47H-abd	N47H-abf
Chl *a*	8.0 ± 0.1	3.9 ± 0.3	6.4 ± 0.2	8.1 ± 0.1	5.7 ± 0.2	6.5 ± 0.1
Chl *b*	4.0 ± 0.1	4.5 ± 0.2	4.2 ± 0.2	3.9 ± 0.1	4.3 ± 0.1	4.0 ± 0.0
Chl *d*		3.6 ± 0.5			2.0 ± 0.3	
Chl *f*			1.3 ± 0.0			1.5 ± 0.0
Chl/Car	4.9 ± 0.2	5.7 ± 0.8	5.2 ± 0.3	5.1 ± 0.1	5.0 ± 0.1	5.4 ± 0.1

a The pigment content is normalized to 12 Chls per complex. The values are the averages reported for two reconstitution experiments each with two technical replicas.

### 3.3 Chl *d* is Involved in the Red-Forms

**Figure 3:**
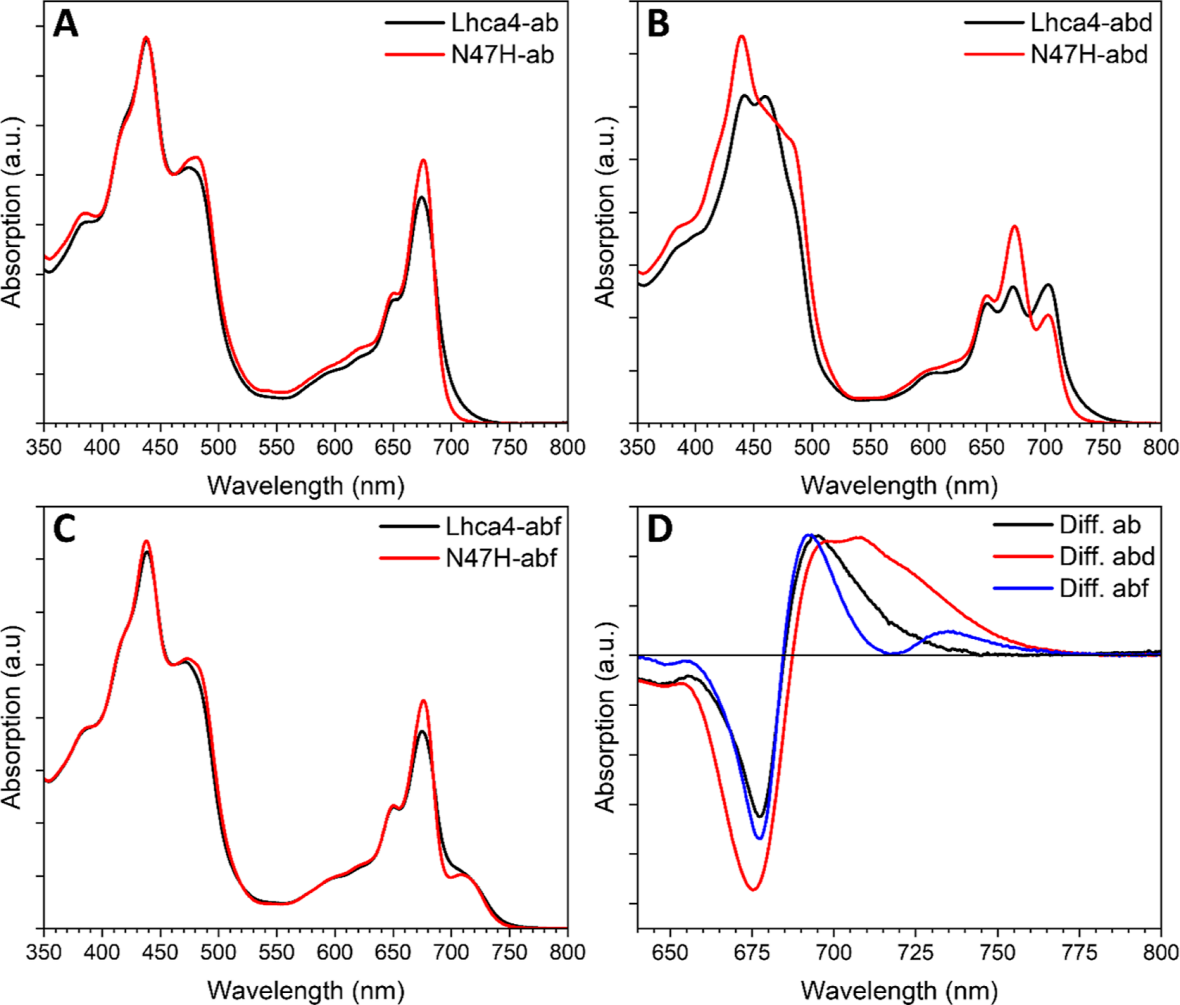
Comparison of the RT absorption spectra of Lhca4 and N47H complexes. Absorption spectra of Lhca4-ab and N47H-ab (A), Lhca4-abd and N47H-abd (B) and Lhca4-abf and N47H-abf (C). (D) Difference absorption spectra in the Chl Q_*y*_ region of the Lhca4 complexes minus their N47H mutants. The spectra are normalized to their area in the Q_*y*_ region (λ = 630–800 nm) scaled to the Chl content as retrieved by the pigment composition analyses (see Table 1).

The mutation N47H is known to abolish the interactions that give rise to the red-forms in Lhca4.^3^ Comparison of this mutant to its WT thus allows for extraction of the features of these spectral forms. In Figure 3 the absorption spectra of Lhca4 and N47H reconstituted with *a* + *b* (Figure 3A), *a* + *b* + *d* (Figure 3B) and *a* + *b* + *f* (Figure 3C) are shown. The loss of red-forms in N47H-ab is clearly observed as a significant decrease in far-red absorption and a concomitant gain around 675 nm (Figure 3A), as previously reported.^3^ A very similar feature is observed when Lhca4-abf and N47H-abf are compared (Figure 3C), indicating that the same red-forms are present in Lhca4-*abf*. This can be more easily appreciated by looking at the Lhca4 minus N47H difference absorption spectrum for the *ab* and *abf* complexes (Figure 3D). Notably, a small positive feature peaking at 735 nm is visible in the difference spectrum. This could indicate that in a small fraction of the Lhca4-abf complexes, a red-shifted Chl *f* form is present. For the samples containing Chl *d* the comparison of the absorption spectra reveals a loss of the red-most shoulder in the N47H mutant (Figure 3B) that extends up to 760 nm, demonstrating that Chls *d* are present in the 603 and/or 609 sites in Lhca4-*abd*.

### 3.4 Lhca4 RT Emission Suggests Promiscuous Chl *f* Binding

**Figure 4:**
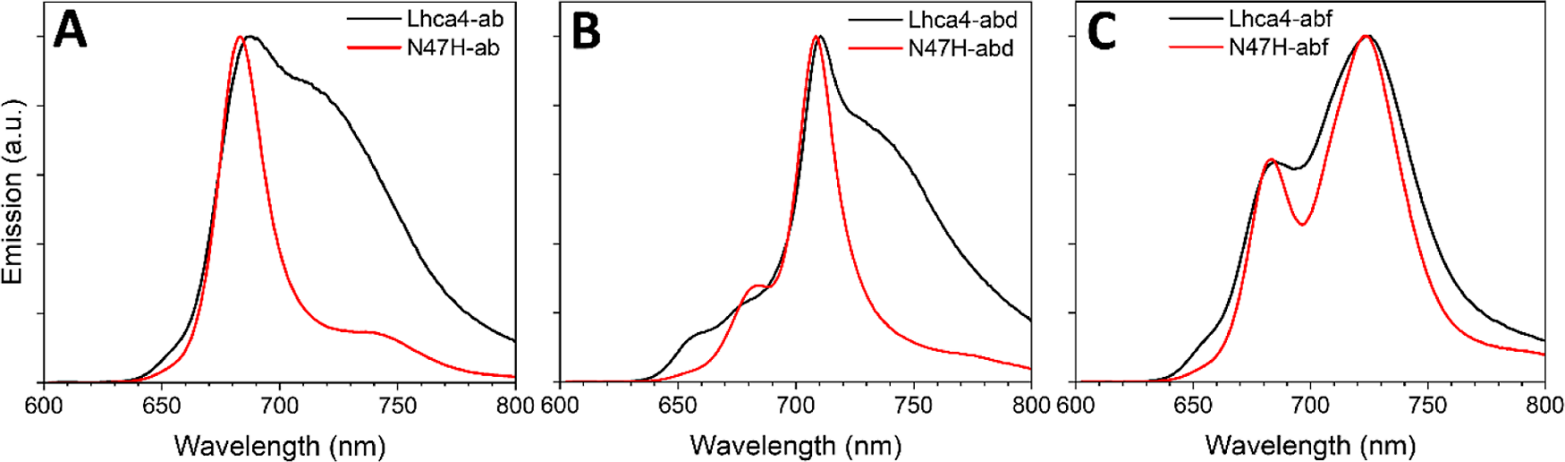
Comparison of the RT emission spectra of the Lhca4 and N47H complexes. Emission spectra of Lhca4-ab and N47H-ab (A), Lhca4-abd and N47H-abd (B) and Lhca4-abf and N47H-abf (C). The samples were excited at 500 nm, and the spectra are normalized to their maxima.

The Lhca4 RT emission spectrum shows a peak around 680 nm with a large shoulder that extends toward the far-red (Figure 4A). In N47H-ab, the red-form absorption is abolished, and consequently the far-red shoulder is absent in the emission spectrum, which more closely resembles that of an Lhcb (Figure 4A). A similar behavior is observed for the complexes containing Chl *d*: Lhca4-abd shows a blue peak with a red shoulder that is absent in N47H-abd (Figure 4B). The emission spectra of Lhca4-abf and N47H-abf have the same maxima but in N47H the emission profile is narrower. Furthermore, the blue peak in Lhca4-abf is at the same position as the blue peak in Lhca4-ab (Figure 4A,C). The presence of a significant contribution of Chl *a* in the abf emission spectra (around 680 nm) indicates that not all complexes in the ensemble contain Chl *f*. This implies that there is no single Lhca4 Chl binding site that displays a high binding affinity for Chl *f*. The small shoulders that are present around 650 nm in all samples show the presence of a small portion of disconnected Chls *b*.

### 3.5 Incorporation of Red-Shifted Chls in Lhca4 Does Not Affect the Excited State Decay Kinetics

**Figure 5:**
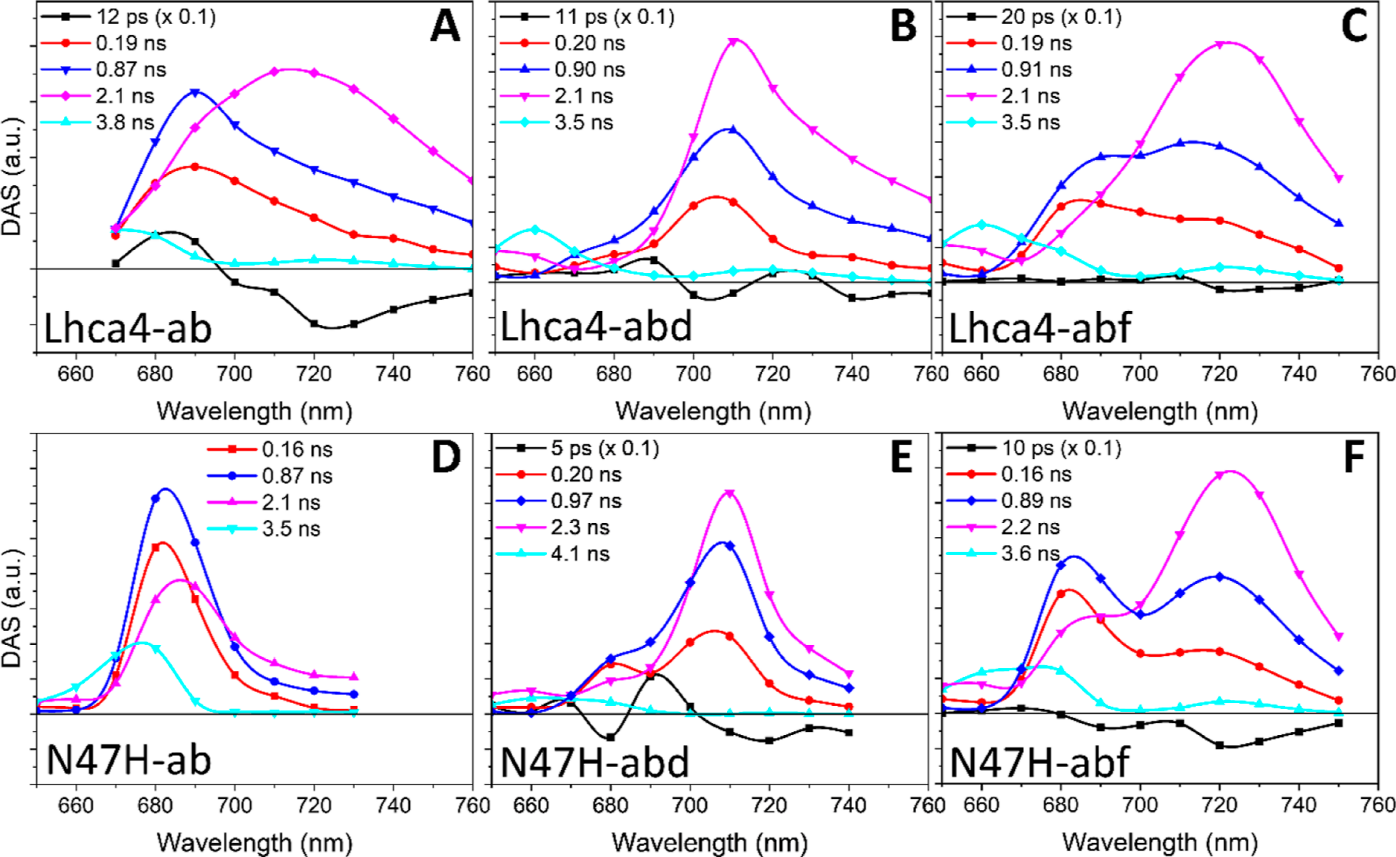
Global analysis results of time-resolved fluorescence measurements of the Lhca4 and N47H complexes. Decay-associated spectra (DAS) of Lhca4-ab (A), Lhca4-abd (B), Lhca4-abf (C), N47H-ab (D), N47H-abd (E), and N47H-abf (F).

To study the effect of the incorporation of Chl *d* and Chl *f* on the excited state decay of Lhca4, we performed time-resolved fluorescence measurements. The results of the global analysis of the data are listed in Figure 5. Five components are needed to accurately fit the data of Lhca4-ab (Figure 5A). The first 12 ps DAS (Figure 5A, black) shows the typical +/- feature of an energy transfer component. The decay has three main components of 0.19 ns (Figure 5A, red DAS), 0.87 ns (Figure 5A, blue DAS) and 2.1 ns (Figure 5A, magenta DAS). The two shorter components show a higher amplitude in the blue part of the spectrum, whereas the longer one peaks in the far-red. This was shown to be an intrinsic feature of Lhca4 and it is due to distinct conformations.^26^ The last small component with a lifetime of 3.8 ns (Figure 5A, cyan) is attributed to disconnected Chls, as its maximum is blue-shifted compared to the other DAS and since Chl in solution have an excited state lifetime of >3.5 ns. The decay in N47H-ab can be described with virtually the same lifetimes of 0.16, 0.87, and 2.1 ns (Figure 5D), but spectrally, these components lack amplitude in the far-red, which is consistent with previous results.^26^

A similar picture arises for Lhca4-abd (Figure 5B): the energy transfer component has a lifetime of 11 ps and shows transfer to different Chl pools at 705 and 740 nm. Three components describe the decay. Their lifetimes are almost identical to those in Lhca4-ab (0.20 vs 0.19 ns, 0.90 vs 0.87 ns, and 2.1 ns vs 2.1 ns), but the spectra are all red-shifted, showing maxima above 700 nm. The maximum of the spectrum redshifts with increasing lifetime and the 0.19/0.20 ns component shows the smallest amplitude in the far-red region (Figure 5A,B, red DAS). The 3.5 ns component mainly describes the decay of disconnected Chls (Figure 5B, cyan DAS). The situation in N47H-abd is very similar, apart from the decrease in amplitude in the far-red of all decay components (see Figure 5E and in particular the magenta DAS). The relative areas of the DAS of the three main decay components are also comparable in Lhca4-ab and Lhca4-abd (∼0.2 ns:∼0.9 ns:∼2.0 ns = 48:34:18 and 50:33:17 in Lhca4-ab and Lhca4-abd, respectively). These results demonstrate that the lifetimes and relative abundances of the different conformations in the ensemble are not affected by the incorporation of Chl *d*.

The same general trend applies to Lhca4-abf: the main decay components have very similar lifetimes and possess increasing amplitude in the far-red region with increasing lifetimes (Figure 5C). The involvement of Chl *f* is visible as a shoulder in the far-red, especially in the two shorter decay components. The relative amplitude of the three decay components is again very similar to Lhca4-ab and Lhca4-abd (∼0.2 ns:∼0.9 ns:∼2.0 ns = 47:34:18). The N47H-abf mutant differs in the sense that all decay components still show large amplitude in the far-red, but they also possess relatively more amplitude on the blue side of the spectrum, which confirm that the red most emission originates from canonical Chls *a* red-form while the Chls *f* occupy other binding sites. The presence of the emission around 680 nm in both the Lhca4-abf and N47H-abf samples shows that not all complexes in the ensembles contain Chl *f*. The large spectral separation between the complexes with and without Chl *f*, and the comparison of Lhca4 and Lhca4-N47H, allows appreciating the features of the complexes with Chl *f* and those additionaly containing the Chl *a* red-forms. This comparison shows that the same general trend applies to all complexes.

### 3.6 Excitation Energy Transfer Involving Chls *d* and *f* in Lhca4

To study the effect of Chls *d* and *f* on the energy transfer pathways and kinetics of the complexes, we performed ultrafast transient absorption measurements at RT. The excitation wavelength was set at 642 nm in all experiments to preferentially excite Chls *b*. The spectrotemporal maps of the data are presented in Figure S5.

A quantitative picture of the energy transfer dynamics was obtained by globally analyzing the data. The fits are all excellent and are presented in Figures S6–S11. All data sets were globally fitted using six components. Models with six components yielded in all cases better fits than those with five, and all components display a distinct structure. Models with seven components, however, either could not be globally fitted or yielded shapeless spectra that resemble noise.

**Figure 6:**
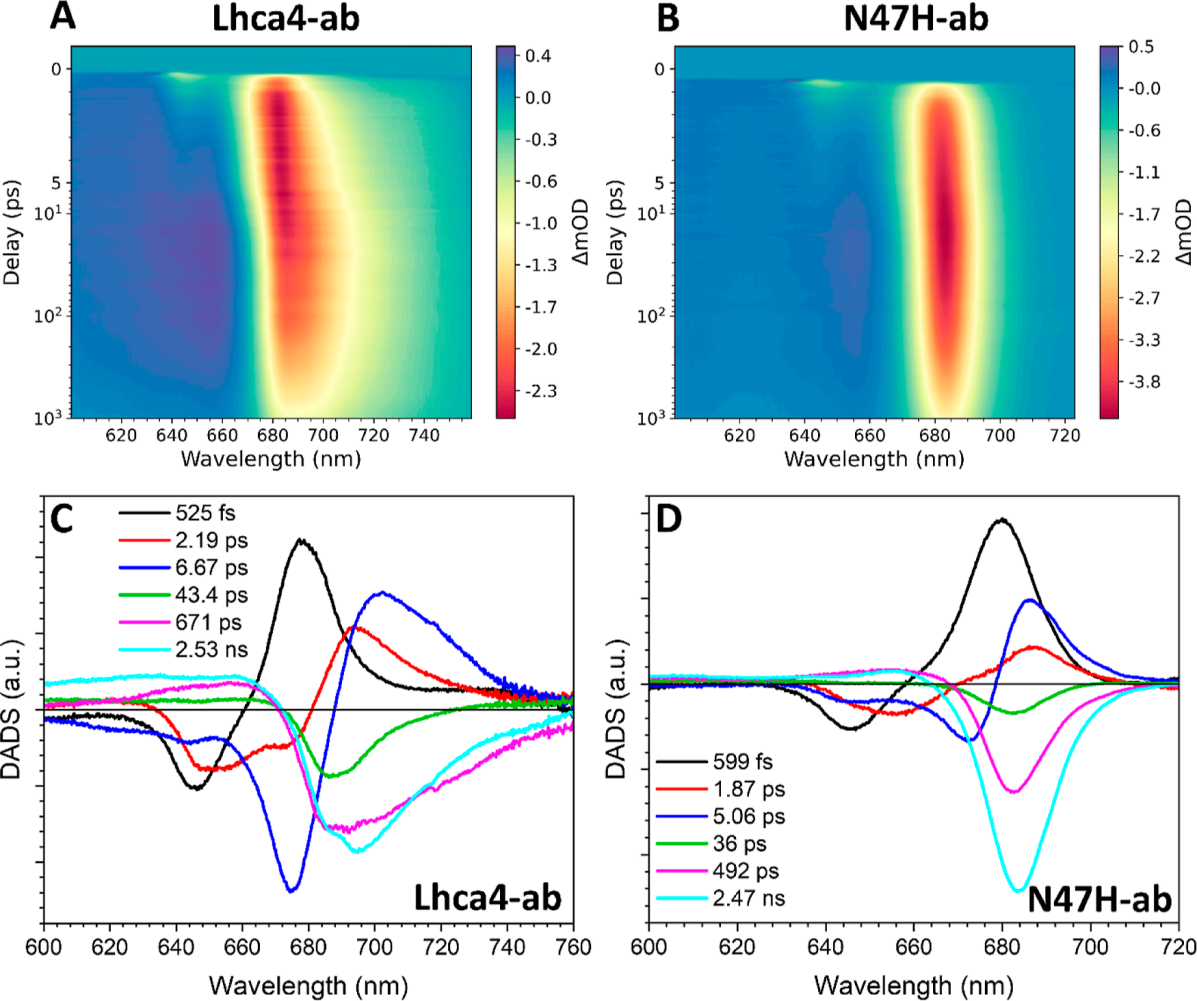
Transient absorption data for Lhca4/N47H-ab excited at 642 nm. Spectrotemporal maps of Lhca4-ab (A) and N47H-ab (B). DADS of Lhca4-ab (C) and N47H-ab (D).

We first present the results of Lhca4-ab and N47H-ab (Figure 6A,B), using them to frame our discussion on samples containing Chl *d* and *f*. Comparison of the maps clearly shows the contribution of the red-form to the dynamics, which can be appreciated in the Lhca4-ab map as a gradual red-shift of the ground-state bleaching/stimulated emission (GSB/SE) within the first ∼5 ps and a subsequent decay of GSB/SE in the far red region on the order of several hundreds of picoseconds to several nanoseconds. Such dynamics are absent in N47H-ab. The first DADS shows spectral evolution within 525 fs (Figure 6C, black). It has the typical -/+ pattern indicating energy transfer from Chls *b* peaking at 646 nm, to Chls *a* at 677 nm. The second DADS (Figure 6C, red) with a time constant of 2.19 ps shows energy transfer from Chls *b* at 652 nm and Chls *a* at 672 nm to a mix of low energy Chls *a* and red-forms, as the positive feature peaks (694 nm) in between these states.^8^ The next evolution takes place in 6.67 ps (Figure 6C, blue DADS) and concerns the last energy equilibration process, which involves Chls *b* at 644 and Chl *a* at 675 nm as energy donors and mainly the red-forms, as acceptors. The final three DADS are negative in the Q_*y*_ region and represent excited-state decays. The shortest component (43.4 ps) (Figure 6C, green) has a smaller amplitude in the far red than the longer ones (671 ps and 2.53 ns) (Figure 6C, magenta and cyan), which is consistent with the time-resolved fluorescence measurements (Figure 5A and see above). The decay components are better resolved in the TCSPC measurements due to a longer time window and a superior signal-to-noise ratio. The transient absorption measurements, on the other hand, have a far superior time and spectral resolution that allow us to follow the energy transfer steps. Below we therefore mainly discuss the energy transfer processes.

Six components were also needed to fit the data of N47H-ab (Figure 6D). The lifetimes of the three energy transfer components are similar to those observed in Lhca4 (599 vs 525 fs, 1.87 vs 2.19 ps, and 5.06 vs 6.67 ps). Spectrally, the energy transfer DADS in N47H-ab notably lacks spectral evolution in the region where the red-forms absorb. However, it can be concluded that the red-forms do not significantly slow down the energy equilibration in Lhca4. Similar to the case of Lhca4-ab, the excited state decay is described by the final three components of 36 ps (green), 492 ps (magenta), and 2.47 ns (cyan). These components have similar spectra, although the two longer ones do show relatively more amplitude in the red, consistent with the TSCPC data (Figure 5D).

**Figure 7:**
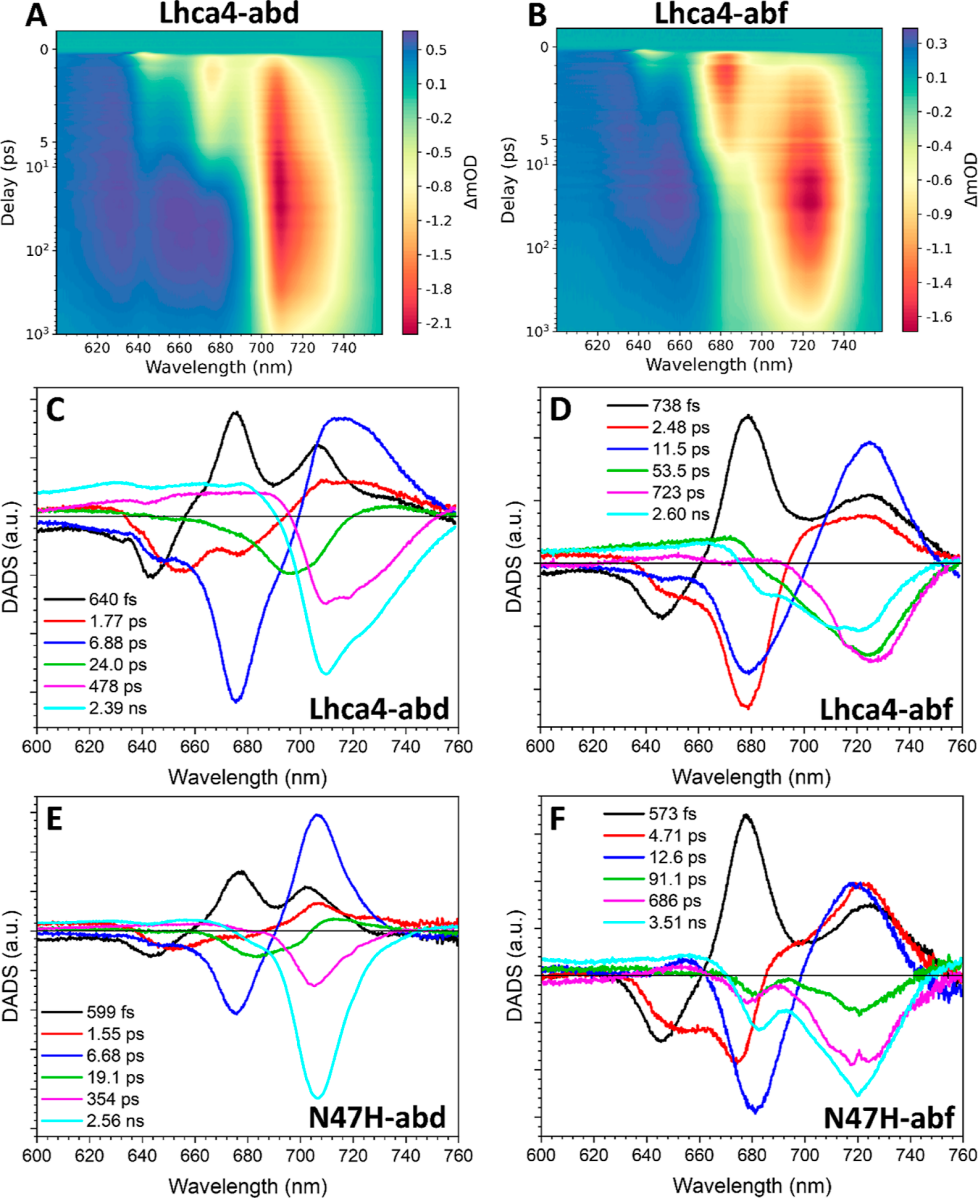
Transient absorption measurements of complexes containing Chls *d* and *f*. Spectrotemporal maps of Lhca4-abd (A) and Lhca4-abf (B). DADS of Lhca4-abd (C), Lhca4-abf (D), N47H-abd (E), and N47H-abf (F).

The incorporation of Chls *d* and *f* in Lhca4 has a large effect on the spectrotemporal evolutions as can be appreciated from the TA maps of Lhca4-abd and abf (Figure 7A,B). In Lhca4-abd the first DADS (Figure 7C, black) with a time constant of 640 fs describes the transfer of about half of the energy initially residing on the Chls *b* (which peak at 643 nm) to Chls *a* at 675 nm and *d* at 706 nm. A small positive shoulder extending around 735 nm is also present in this DADS, indicating that the Chl *d* red-forms are already populated within this time frame. The second DADS (1.77 ps, Figure 7C, red) describes the transfer from Chls *b*, peaking at 654 nm, and Chls *a* at 676 nm. The positive signal for the acceptor states shows a mixture of Chls *d* and Chl *d* red-forms, which is equivalent to the second DADS in Lhca4-ab (Figure 6D, red). The third DADS (6.88 ps, Figure 7C, blue) represents the slow transfer from Chls *b* at 648 nm and Chls *a* at 676 nm. The positive signal peaks at 714 nm and has a broad shoulder extending into the far-red, indicating the involvement of the Chl *d*-red-forms as energy acceptors. The fourth DADS, (24.0 ps, Figure 7C, green), mainly describes the decay to the ground state for the fast/blue conformation but also contains some small positive amplitude around 735 nm, which accounts for the slow transfer to the Chl *d* red-forms. The rest of the decay is described with the last two DADS of 478 ps and 2.39 ns (Figure 7C, magenta and cyan, respectively), which peak at 708 nm and contain significant amplitude in the region of the Chl *d* red-forms, consistent with the TCSPC data (Figure 5B).

To separate the contribution of the Chl *d* red-forms in the dynamics, a comparison can be made between the EET kinetics in Lhca4-abd (Figure 7C) and N47H-abd (Figure 7E). The three pure energy transfer components resolved for N47H-abd have time constants of 599 fs, 1.55 ps, and 6.68 ps, which are comparable to those of Lhca4 (640 fs, 1.77 ps, and 6.88 ps). The fourth DADS (19.1 ps, Figure 7E, green) is a mixture between an energy transfer component and a decay component, but here the band is blue-shifted, peaking at 713 nm. The difference between WT and mutant is analogous to the case of Lhca4/N47H-ab and shows that the energy equilibration processes in Lhca4-abd are not slowed down by the Chl *d* red-form. The decay is described by the two final DADS of 354 ps and 2.56 ns (Figure 7E, magenta and cyan) that, at variance with the Lhca4-abd decay, lack the amplitude in the region of the Chl *d* red-forms.

Next, we discuss the results for the complexes binding Chl *f*. The 1–2 Chls *f* per complex (Table 1) significantly affect the dynamics (see Figure 7B). The first DADS (Figure 7D, black) of Lhca4-abf shows that within 738 fs 70% of the energy that was initially on the Chls *b* (peak at 646 nm) is transferred to Chls *a* that peaks at 678 nm. In this time scale, there is also some significant transfer to red-shifted species (red-form/Chl *f*). Compared to the first Lhca4-ab DADS (Figure 6D, black), which has a similar time constant (525 fs), the amplitude at around 725 nm is far larger. This suggests that most of the energy in this early step is transferred to Chl *f*. This agrees with the observation that the first DADS in N47H-abf is very similar (Figure 7F, black, τ = 573 fs). The second DADS (2.48 ps, Figure 7D, red) describes the transfer of a quarter of the energy initially on Chls *b* (peak 653 nm) and most of the energy transfer from Chls *a* (peak at 678 nm). The acceptor states are a mix between low energy Chls *a*, Chl *a* red-forms and Chls *f*. Compared to the second DADS of Lhca4-ab (Figure 6D, red, τ = 2.19 ps) there is again a larger amplitude around 725 nm, which shows that also at this time scale Chl *f* is an energy acceptor. In N47H-abf the second DADS (Figure 7F, red) is more than twice as long as that in Lhca4-abf (4.71 ps). It shows energy transfer from the same species (Chls *b* at 653 nm and Chls *a* at 678 nm), but the positive amplitude around 724 nm is enhanced. The third Lhca4-abf DADS (Figure 7D, blue) has a lifetime significantly longer (11.5 ps) than the corresponding DADS in Lhca4-ab and Lhca4-abd (6.67 and 6.88 ps, respectively). It mainly describes transfer from Chls *a* peaking at 678 nm to Chl *f*/Chl *a* red-forms at 725 nm. Considering that the final energy equilibration step in Lhca4-ab is much faster (6.67 ps), it must be that the transfer to Chl *f* is responsible for slowing down the energy equilibration in the complex. Indeed, a slow EET component of 12.6 ps, which shows a significant transfer to Chls *f* is also present in the N47H complex (Figure 7F, blue).

The decay is described in both samples with the last three components, which have lifetimes of 53.5 ps, 723 ps, and 2.60 ns in Lhca4-abf (Figure 7D, green, magenta, and cyan) and of 91.1 ps, 686 ps, and 3.51 ns in N47H-abf (Figure 7F, green, magenta, and cyan). Consistent with the emission spectra (Figure 4C) and the TCSPC data (Figure 5C,F) part of the decay arises from complexes that do not contain Chl *f* and show significant amplitude in the region of Chl *a* around 680 nm. To isolate the kinetics of the complexes that do contain Chl *f*, a target analysis was performed using two parallel schemes: the sequential scheme that resulted from the global analyses on the Lhca4/N47H-ab samples and an unconstrained sequential scheme (see the Experimental Section). To focus on the EET dynamics, only the first 100 ps were fitted, and as a result, the decay could be described with a single component. The relative abundance of complexes with Chl *f* was a free-fitting parameter in the analyses. For the Lhca4-abf sample, we found that 67% of the complexes contained Chl *f*, whereas for the N47H sample this value was 62%, which in first approximation is consistent with their emission spectra (Figure 4C). The corresponding DADS of the sequential scheme for the complexes with Chl *f* for the Lhca4-abf and N47H-abf data set are shown in Figure S13. The full sets of EADS and full kinetic schemes of the target analyses are also shown in Figure S13. The fitting results are shown in Figures S14 and S15.

Three energy transfer DADS were resolved for the complexes containing Chl *f* in the Lhca4-abf sample (Figure S13A, black, red, and blue). The first DADS has a time constant of 605 fs (Figure S13A, black), and is similar in shape and in time constant as the first DADS of the Lhca4-abf global analysis (Figure 7D, black). The main differences are in the Chl *b* peak that shows minima at 638 and 644 nm (instead of 646 nm) and in the amplitude of the Chl *a* peak at 676 nm, which is significantly reduced. The second DADS (Figure S13A, red) has a longer time constant than its counterpart from the global analysis (4.17 ps vs 2.48 ps) and has a sharper spectrum in the region of Chl *f*/Chl *a* red forms (see Figure 7D, red), showing that Chl *f* is populated at a slower rate. The final EET DADS (Figure S13A, blue) is substantially longer than in the global analysis (Figure 7D, blue, 18.7 vs 11.5 ps). The component shows a broad negative peak extending beyond 700 nm, and positive amplitude peaking at 724 nm. The extension of the negative amplitude beyond 700 nm might indicate excitation energy equilibration between the Chl *a* red-forms or a relatively blue Chl *f* and (red) Chl *f* on this time scale. The final component (Figure S13A, magenta) does not show any decay of canonical Chl *a* GSB/ESA around 680 nm, showing that the target model effectively describes the Chl *f*-containing complexes.

Also the Chl *f*-containing complexes in the N47H-abf sample could be described with three EET components (Figure S13B, black, red, and blue). The first DADS (Figure S13B, black) has a similar time constant and spectrum as its global analysis counterpart (Figure 7F, black, 586 fs versus 573 fs), except that the positive amplitude around 676 nm is significantly reduced and the negative region for the donating Chls *b* has a broader profile and shows a shoulder around 650 nm. The second DADS (Figure S13B, red) also has a similar time constant as in the global analysis (4.83 ps versus 4.71 ps). In terms of shape, this DADS (Figure S13B, red) lacks negative amplitude around 650–660 nm and positive amplitude around 690 nm, indicating that those components in the global analysis (Figure 7F, red) are mostly associated with complexes without Chl *f*. The final DADS (Figure S13B, blue) has a very similar lifetime and shape as the global analysis DADS (Figure 7F, blue, 13.9 vs 12.6 ps), already showing that in the global analysis this component could be solely ascribed to Chl *f*-containing complexes, as expected. The final DADS (Figure S13B, magenta) again shows the decay of just the complexes with Chl *f*, as evidenced by the lack of negative amplitude around 680 nm.

## 4 Discussion

### 4.1 Competitive Binding of Chlorophyll *d* and *f* to Lhca4

The experiments performed in this study shed light on the competitiveness of different Chls for binding to Lhca4 and how their presence impacts the functionality of the complex. First, the number of Chls *b* bound to Lhca4 does not differ much between complexes (3.9–4.5 Chls *b*/Lhca4, Table 1), indicating a strong affinity for Chl *b* for some sites. This is analogous to the situation of LHCII, which possesses several sites that exclusively bind Chl *b*, the occupation of which is necessary for the assembly of the complex.^47−49^ This restricts the competitive binding of Chl *d* and *f* to Chl *a* sites. Lhca4 binds Chl *a* and *d* to a similar extent (Chl *a*/*d* = 1.08), while it has a lower affinity for Chl *f*, which is outcompeted by Chl *a* (Chl *a*/*f* = 4.9).

It is interesting to note that the N47H mutation induces a loss of 1.6 Chls *d*, which are replaced by Chls *a* (see Table 1). This is a strong indication that in Lhca4-abd Chl *d* occupies site 603 and that the mutation of the axial ligand of this site from N to H changes its occupancy to Chl *a*. Inspection of the crystal structure of Lhca4^25^ shows that a hydrogen bond can be formed between F215 and the formyl group of a Chl *d*, when this chlorophyll has the same orientation. This organization is likely compromised in the N47H mutant due to the bulkier ligand that would push the Chl *d* formyl group away from F215. To quantify the strength of this hydrogen bond, we performed DFT calculations and found a value of −3.86 kcal/mol, which might explain the Chl *d* selectivity of the 603 site. These results suggest that the inclusion of a residue capable of forming H bond with the formyl group of Chl *d* (and presumably Chl *f*) may effectively shift the affinity of a binding site toward these Chls. This then represents a practical approach for engineering novel light-harvesting complexes with an enhanced capacity to bind far-red absorbing pigments.

### 4.2 Environmental Factors Fine-Tune the Transition Energy of the Chls

The complexes containing Chl *f* absorb further into the far-red than Lhca4-ab (see Figure 2A), but surprisingly their 77 K emission spectrum peaks at shorter wavelengths and is narrower than that of Lhca4-ab (Figure 2B). This puzzling observation can, in principle, be due to the presence of two, spectrally distinct, conformations of the complexes, one with “blue” and the other with “red” emission, where the “red” one is more quenched in the Chl *f* complexes than the “blue” one. However, although time-resolved data (Figure 5) show the presence of different conformations, they also demonstrate that the “red” conformation is associated with lifetimes longer than those of the “blue” one, in all complexes, including those containing Chl *f*. To understand the origin of the apparent mismatch between absorption and fluorescence, we performed a Gaussian deconvolution of the 77 K absorption spectra of the complexes containing Chl *a* and *b* and those additionally containing Chl *f* (Figure S16). The analysis revealed that the Chl *a*-red-forms, which are responsible for the emission of Lhca4-ab, are present also in Lhca4-abf and they have practically the same broad spectrum as in Lhca4-ab. This means that also in Lhca4-abf, sites 603 and 609 are occupied by Chls *a*. Lhca4-abf shows an additional prominent absorption band, peaking around 712 nm, with a fwhm comparable to that of the bulk Chls *a* (the fwhm of the Chl *f* band is even slightly larger as a consequence of its higher oscillator strength^29^). This absorption form is also present in the N47H mutant and thus can be attributed to Chls *f* in sites other than 603 and 609. The very large Stokes shift of the red-forms^6,8^ explains why the low-temperature emission spectrum of Lhca4-ab peaks at longer wavelengths than that of N47H-abf (in which the terminal emitter is a Chl *f*, but there are no red-forms, Figure S4). The same is true about the width of the emission band in these complexes: in Lhca4-abf it is narrower than that in Lhca4-ab and broader than that in N47H-abf (see also the RT emission spectra in Figure 4). This is because while in Lhca4-ab the red-forms are the most populated at equilibrium, in Lhca4-abf both the Chl *a*-red forms and the Chl *f* forms are substantially populated, and thus both contribute to the emission. As an estimate for the 0–0 transition energy of Chl *a* red forms and Chl *f*, we have taken the intersection point between their low-temperature absorption and emission spectra. The absorption spectra were retrieved from the Gaussian deconvolution (Figure S16). For the emission spectrum of the red-form we took the Lhca4-ab spectrum (which contains the red-form and lacks Chl *f*) and for the Chl *f* spectrum, the N47H-abf emission spectrum (which contains Chl *f* and lacks the red-form). For the red-forms we then find the intersection to be at 715.6 nm (13,974 cm^–1^) and for Chl *f* at 718.4 nm (13,920 cm^–1^), showing that these pigments possess comparable transition energies (difference of 0.26 *k*_B_*T* at RT).

In contrast to the case of Lhca4-abf, the data for Lhca4-abd demonstrate that Chl *d* is substituting Chl *a* in the red-form binding sites 603 and/or 609. To extract the features of the Chl *d* red-form, Gaussian deconvolution of the Chl *d*-containing complexes was performed (Figure S16C). The absorption of the Chl *d*-red forms shows a maximum at 721 nm, which is 20 nm red-shifted compared to the Chl *a* red-forms (Figure S16). Notably, the Gaussian deconvolution of the N47H-abd sample (Figure S16D) revealed the presence of a small band peaking at 720 nm, possibly indicating that in this sample the Chl *d* red-form is not completely abolished. The fwhm of the Chl *d* red-form in Lhca4-abd is comparable to that of the Chl *a* red-form in Lhca4-ab (37.7 vs 37.0 nm), and the Stokes shift is identical (29.5 nm). These similarities suggest that the Chl *d* red-forms also result from the mixing between the lowest excited state and a charge transfer state, as in Lhca4-ab.^7,8^ Consequently, it appears that Chls *d* in these binding sites are organized like Chls *a* in Lhca-ab. These observations highlight a potential design principle that can be employed to shift the absorption of far-red pigments even further into the far-red, opening avenues for creating complexes with greatly expanded far-red absorption ranges.

### 4.3 Effect of Chl *d* and *f* on the Energy Transfer Dynamics

The energy transfer dynamics for the Lhca4 complexes are summarized in Table 2. The time constants of the main energy transfer components are very similar in Lhca4-ab and abd, and the species involved are also largely similar, with Chls *d* at 706 nm replacing the Chls *a* 683–687 nm forms and Chl *d* red-forms replacing Chl *a* red-forms (Table 2). Spectral modeling revealed that the 683–687 nm absorption in Lhca4 is mostly associated with Chls 602 and 613, which makes it probable that these sites display a relatively high Chl *d* affinity.^8^

**Table 2:** Lhca4 Energy Transfer Dynamics^a^

	Lhca4-ab		Lhca4-abd		Lhca4-abf	
525–738 fs	*b* 646 nm	*a* 677 nm	*b* 643 nm	*a* 675 nm	*b* 646 nm	*a* 678 nm
				*d* 706 nm		*f* 724 nm
				*d* red-form		*a* red-form
1.55–2.48 ps	*b* 652 nm	*a* 687 nm	*b* 654 nm	*d* 706 nm	*b* 653 nm	*a* 694 nm
	*a* 672 nm	*a* red-form	*a* 676 nm	*d* red-form	*a* 674 nm	*f* 724 nm
	*a* 677 nm					*a* red form
6.67–6.88 ps	*b* 644 nm	*a* 687 nm	*b* 648 nm	*d* 706 nm		
	*a* 675 nm	*a* red-form	*a* 675 nm	*d* red-form		
	*a* 683 nm		*d* 706 nm			
11.5 ps					*a* 681 nm	*f* 724 nm
18.7 ps					*a* red form/Chl *f*	*f* 724 nm

a Summary of the main observed energy transfer lifetimes (main left column) and involved species per complex (last three columns). The left column in each cell represents the energy donor species and the right column shows the accepting species.

In a previous study in which we reconstituted LHCII with the Chls *b* and *d*, we found that the rate of energy equilibration in these complexes is roughly half of that of WT LHCII (containing Chls *a* and *b*) as a result of the smaller overlap integral for Chl *b* → *d*.^15^ This behavior is not observed in Lhca4 containing Chls *a*, *b,* and *d*, which suggests that on average the overlap integrals are not severely diminished in the complex. This is possible if Chls *b* are still closely connected to Chls *a*, which in turn should be closely connected to Chls *d* that are not too distant in energy. The Gaussian deconvolution of the Lhca4-abd complex indeed revealed that there are high energy Chls *d* in the complex (Figure S16C), which can act as intermediary energy acceptors that speed up the excitation energy equilibration. It should also be noted that a reduction in overlap integral could partially be compensated for by the higher oscillator strength of Chl *d* over that of Chl *a*. In any way, no new bottleneck states are introduced in the complex.

In Lhca4-abf the energy transfer dynamics are strongly influenced by the presence of Chl *f* at all time scales, although only ∼1 Chl *f* is bound per complex. This implies that there are multiple energy transfer pathways leading to Chl *f*, potentially because Chl *f* binds promiscuously to multiple sites. Most of the energy transfer to Chl *f* still occurs within 5 ps, which means that in most cases the Chl *f* is well connected with the rest of the Chl manifold. Again, the reduction of the overlap integral as a result of a larger energy separation between Chls *f* and the other Chls in the complex can be at least partially counteracted by the larger oscillator strength of Chl *f*. About one-third of the Chls *f* are populated significantly slower, on the order of ∼12 ps, and this excitation energy originates from Chls *a* absorbing around 681 nm (see Table 2 and Figure 7F). It is possible that in these cases the Chl *f* is located in the relatively more isolated Chl binding sites, such as 604, 613 and 614.^25^

## 5 Conclusions

This work demonstrates that the far-red absorbing Chls *d* and *f* can effectively be incorporated into the PSI antenna Lhca4. Moreover, their absorption can be tuned even more to the far-red by the protein environment which enhances pigment–pigment interactions. This results in complexes with a significantly increased absorption in the far-red region. This is the case even when, as with Chl *f*-containing complexes, only a limited number of sites show affinity for this Chl. This enhancement is also due to the fact that the oscillator strength of the lowest energy band of both Chl *d* and Chl *f* is larger than that of Chl *a*. This property represents an advantage also for excitation energy transfer, as it increases the Förster overlap integral. Moreover, the incorporation of Chl *d* and *f* in Lhca4 does not significantly alter its functional properties: they possess similar features as the Lhca4-ab type regarding excited state lifetime and the energy equilibration within these complexes is ultrafast, both of which are imperative properties for efficient light-harvesting. Furthermore, Chl *d* in Lhca4 can bind to the binding sites responsible for the red-form, resulting in an additional red-shift of 20 nm. These data reveal that the introduction of Chl *d* and *f* into the PSI antenna Lhca4 functionally boosts its absorption toward the far-red, without affecting the transfer rates, suggesting that the incorporation of these Chls into plants could be a promising avenue for improving crop yields. Our findings also provide practical guidelines that could be employed to design proteins able to bind far-red Chls and finetune their absorption spectrum to maximize far-red photon capture.

## Supporting Information Available

Additional experimental data including steady-state optical spectra, transient absorption data (maps, global analyses, fitting quality and power study) and supporting Gaussian deconvolutions of absorption spectra (PDF)
