# Supplementary material for: Coloring Outside the Lines: Exploiting Pigment–Protein Synergy for Far-Red Absorption in Plant Light-Harvesting Complexes
Source: J Am Chem Soc. 2024 Jan 29;146(5):3508–20. doi: 10.1021/jacs.3c13373 (PMC10859958; doi:10.1021/jacs.3c13373)
Supplement: Supplementary file 1 — ja3c13373_si_001.pdf [file ja3c13373_si_001.pdf]

Supporting Information

Coloring Outside the Lines: exploiting pigment-protein synergy for far-red absorption in plant Light-Harvesting Complexes

Eduard Elias[a], Katrin Brache[a], Judith Schäfers[a] and Roberta Croce*[a]

[a] Department of Physics and Astronomy and Institute for Lasers, Life and Biophotonics, Faculty of Sciences, Vrije Universiteit Amsterdam, de Boelelaan 1081, 1081 HV, Amsterdam, The Netherlands.
[*] Corresponding author: r.croce@vu.nl

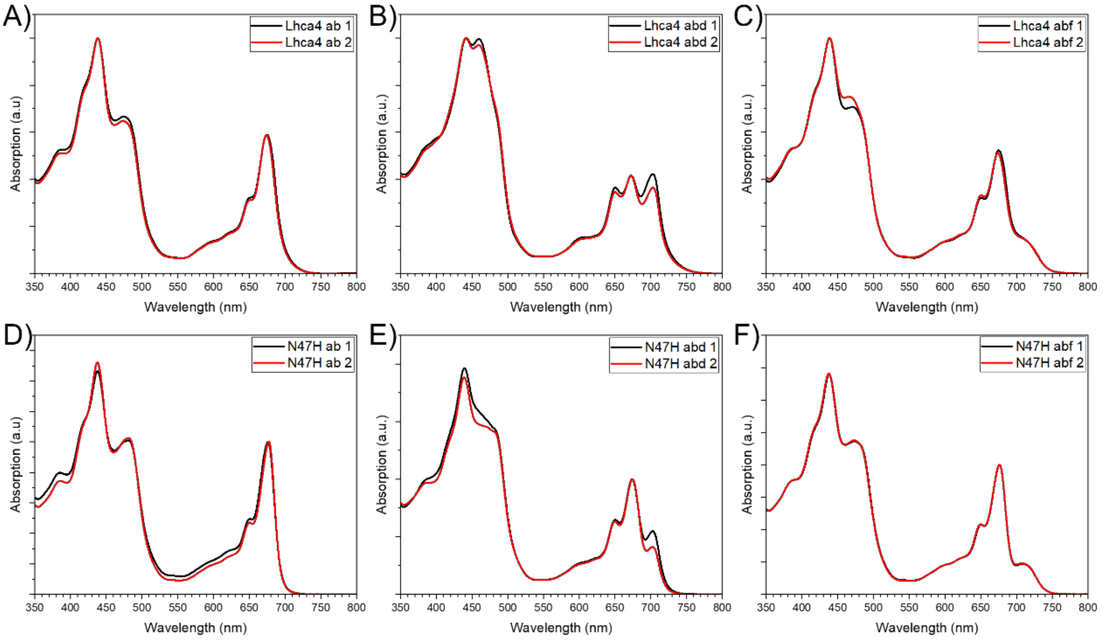

Figure S1. Comparison of RT absorption spectra of the replicas of Lhca4 and N47H. Absorption spectra of the replicas of **(A)** Lhca4-ab, Lhca4-abd **(B)**, Lhca4-abf **(C)**, N47H-ab **(D)**, N47H-abd **(E)** and N47H-abf **(F)** sample.

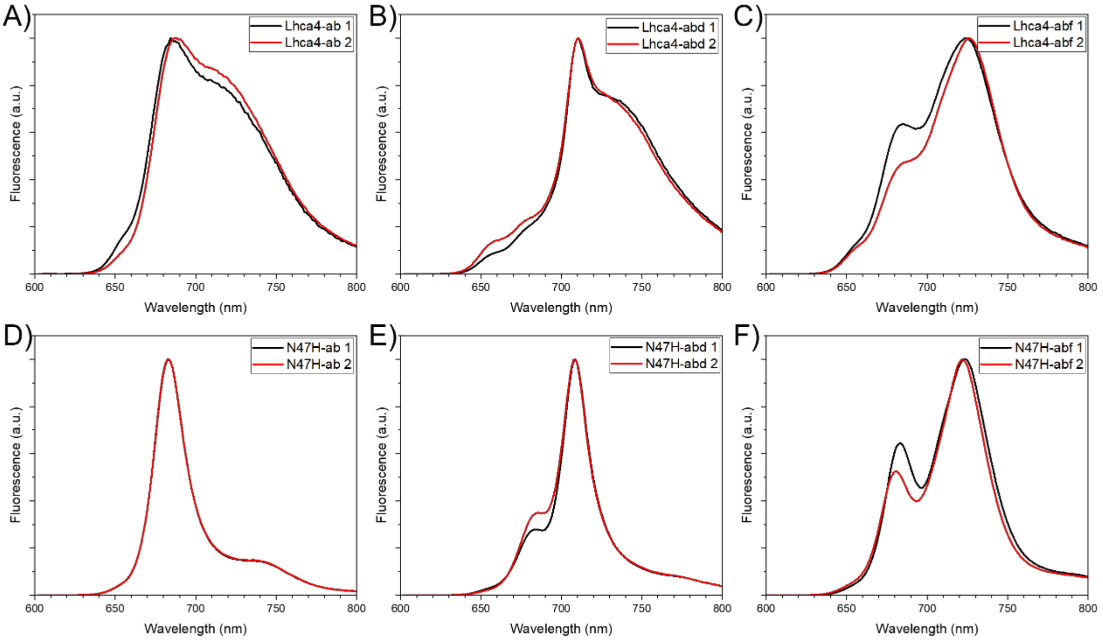

Figure S2. Comparison of RT emission spectra of two replicas of Lhca4 and N47H. Emission spectra of **(A)** Lhca4-ab, Lhca4-abd **(B)**, Lhca4-abf **(C)**, N47H-ab **(D)**, N47H-abd **(E)** and N47H-abf **(F)** sample. Excitation was set at 500 nm.

Text S1.

The emission spectra for the different replicas of the reconstitution experiments (Fig. S2) generally differ more than their corresponding absorption spectra (Fig. S1). This is because in the emission spectra the contribution of the lower energy pigments is strongly enhanced, making differences between them more visible. As a result, small differences in Chl ratios can explain the seemingly large differences in the emission spectra. The Chl *a*/*b* and the Chl *a*/(*d* or *f*) ratios of from the different replicas are shown in Tab. S1. These pigment compositions are in line with the observed behaviour in the emission absorption spectra, e.g. Lhca4-abf 1 has a higher Chl *a*/*f* ratio than Lhca4-abf 2 and the emission spectrum of Lhca4-abf 1 consistently shows more Chl *a* emission than Lhca4-abf 2.

	**Chl *a*/*b***	**Chl *a*/(*d* or *f*)**
**Lhca4-ab 1**	2.88	
**Lhca4-ab 2**	3.03	
**Lhca4-abd 1**	1.26	1.41
**Lhca4-abd 2**	1.33	1.79
**Lhca4-abf 1**	2.44	7.72
**Lhca4-abf 2**	2.18	7.08
**N47H-ab 1**	3.13	
**N47H-ab 2**	3.03	
**N47H-abd 1**	1.96	3.61
**N47H-abd 2**	2.02	5.22
**N47H-abf 1**	2.50	6.73
**N47H-abf 2**	2.44	6.49

Table S1. Chl ratios for the different reconstitution replicas.

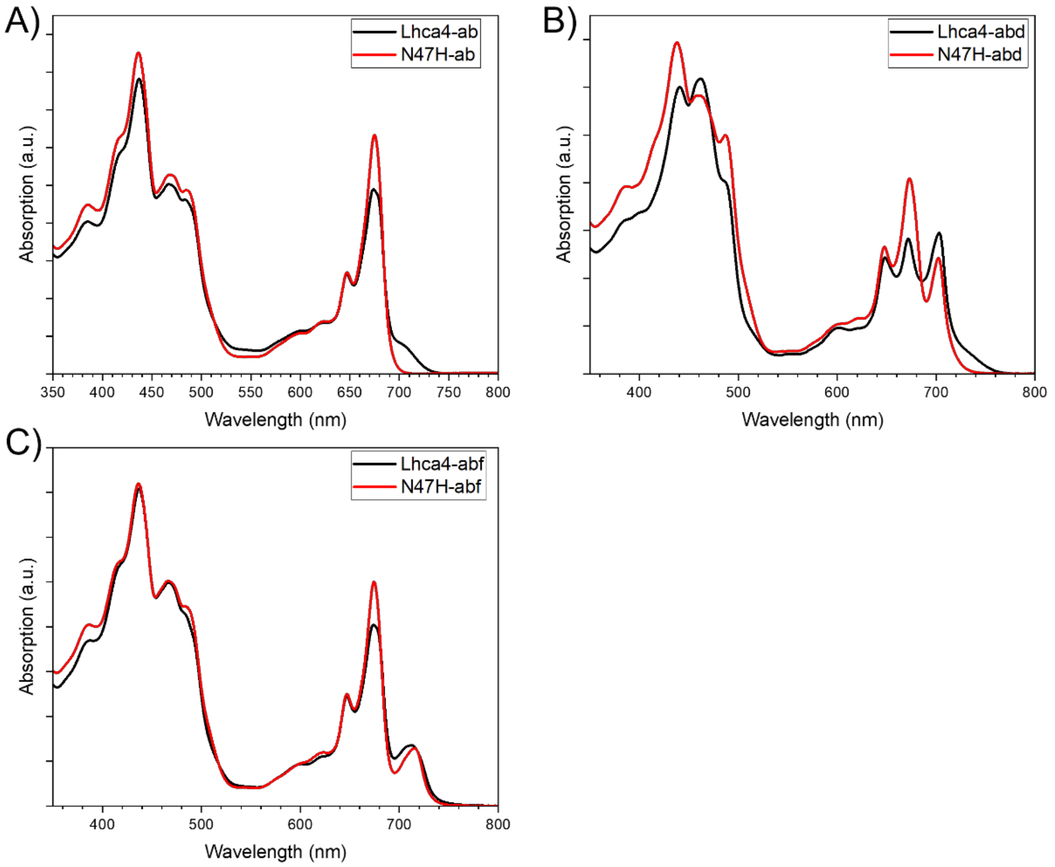

Figure S3. 77 K absorption spectra of Lhca4 and N47H. Comparison of the absorption spectra of Lhca4 and N47H containing Chl *a* and *b* **(A)**, Chl *a*, *b* and *d* **(B)** and Chl *a*, *b* and *f* **(C)**. The absorption spectra are normalised to their area in the Q_y region (λ = 630-800nm) and scaled to their Chl content as retrieved by the pigment composition analyses (see Tab. 1). The relative oscillator strengths Chl *a*:*b*:*d*:*f* were determined to be 1.0:0.67:1.20:1.34 based on their relative absorption in the Q_y region in 80% acetone.

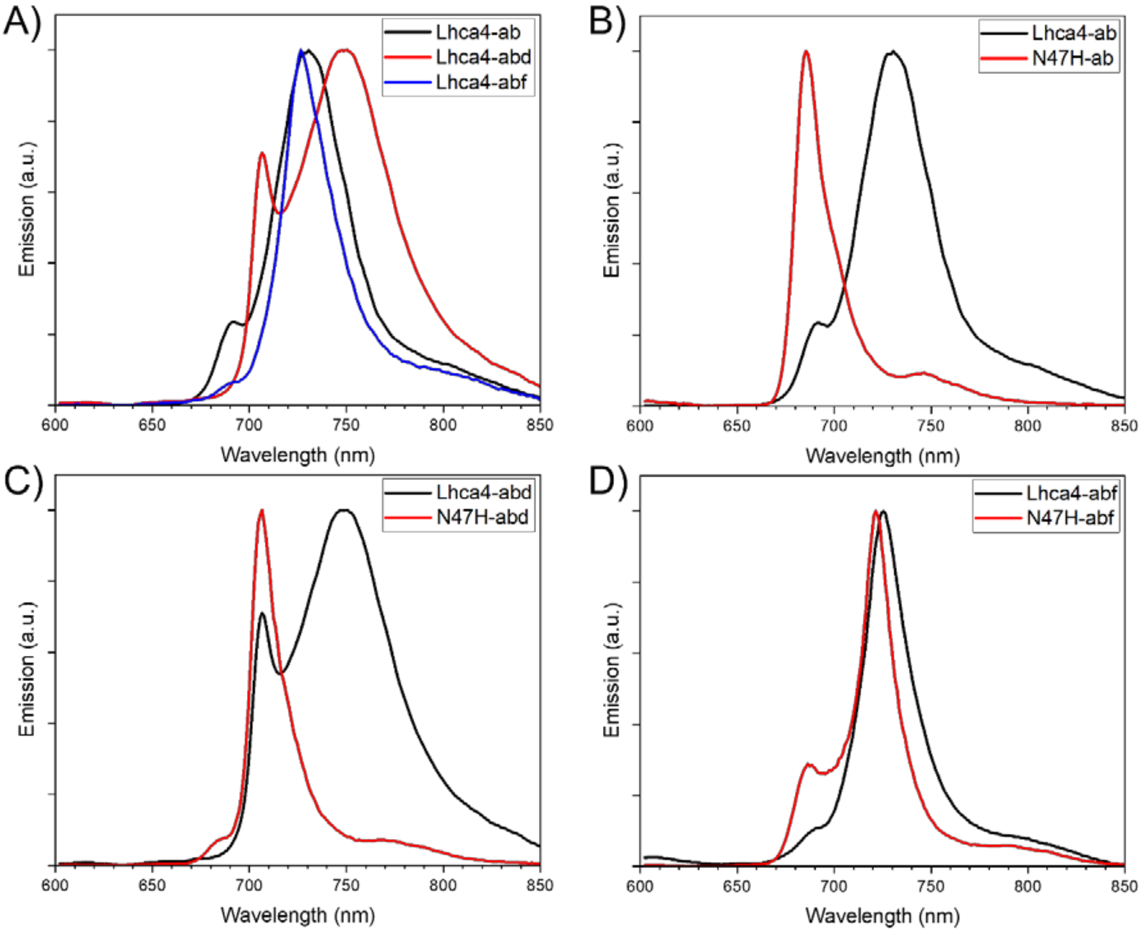

Figure S4. 77 K emission spectra of Lhca4 and N47H. Comparison of the 77 K emission spectra of Lhca4 refolded with different pigments **(A)**. Comparison of the spectra of Lhca4 and N47H containing Chl *a* and *b* **(B)**, Chl *a*, *b* and *d* **(C)** and Chl *a*,*b* and *f* **(D)**. Excitation was set at 500 nm.

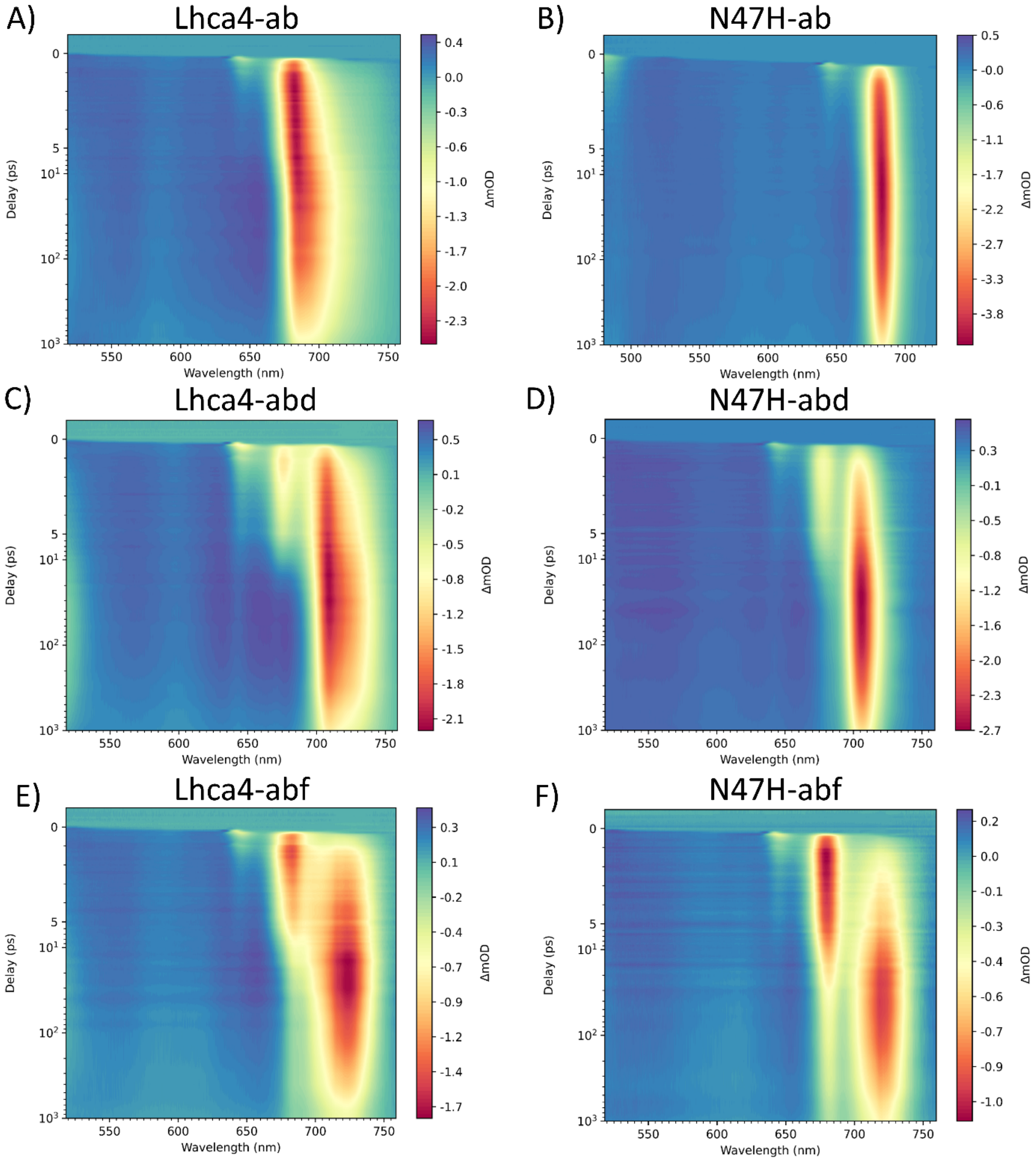

Figure S5. Spectro-temporal transient absorption maps. Spectro-temporal maps of Lhca4-ab **(A)**, N47H-ab **(B)**, Lhca4-abd **(C)**, N47H-abd **(D)**, Lhca4-abf **(E)** and N47H-abf **(F)**. These maps show mainly positive signals in the region λ<630 nm, which can be attributed to the excited-state absorption (ESA) of the Chl molecules. In the Chl Q_y region, the signals are mainly negative and correspond to the ground-state bleach and stimulated emission (GSB/SE) of the excited Chls. Within ~10 ps, rapid decays and rises of GSB/SE are observed, corresponding to the energy equilibration processes in the systems. The total loss of GSB/SE signal in the experiments occurs mostly in the nanosecond time scale and is due to the relaxation to the ground state of the excited Chls.

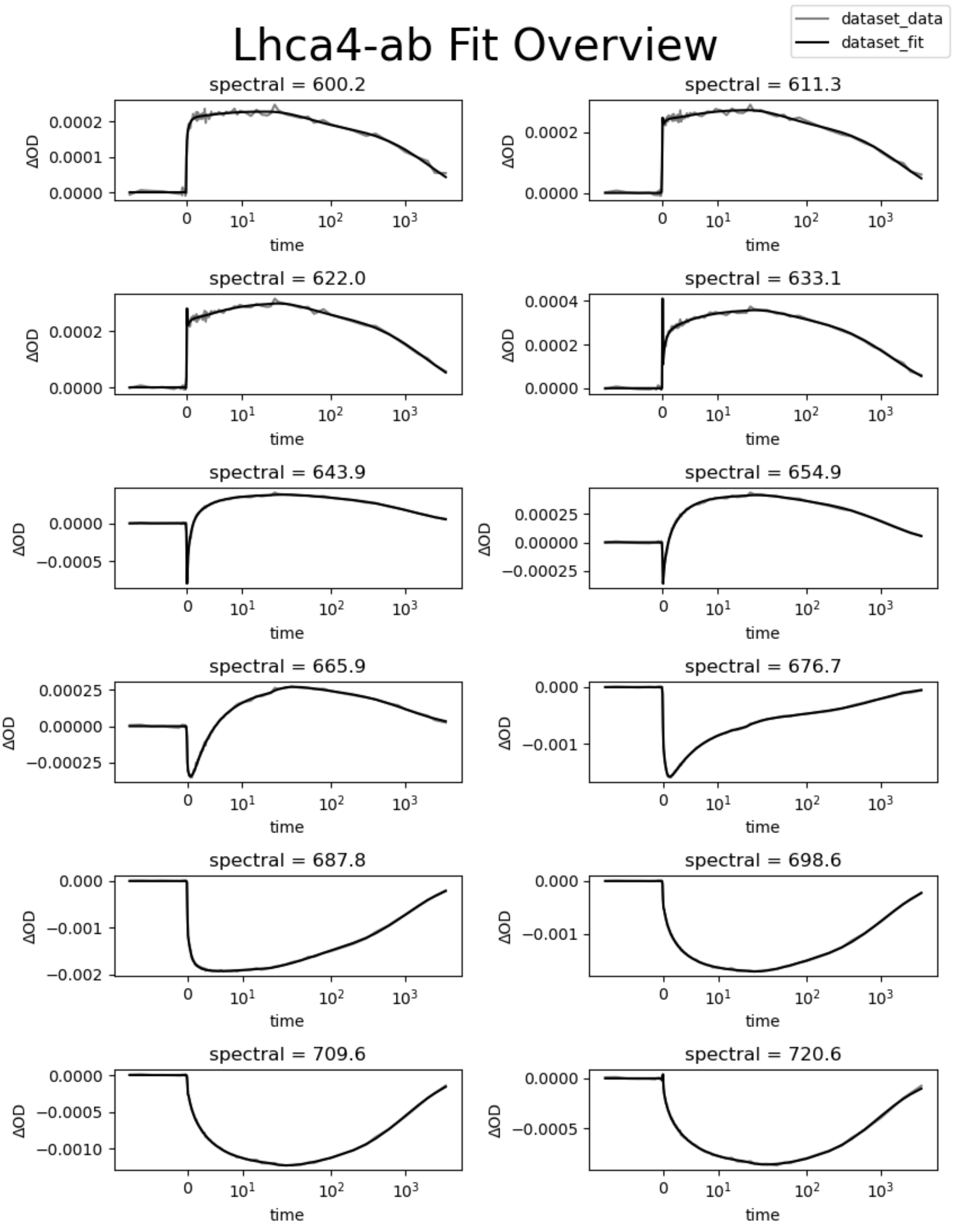

Figure S6. Fitting quality of the global analysis of the Lhca4-ab sample. For a selection of wavelengths the time-trace of the raw data of the transient absorption experiment (grey) is shown along the globally fitted trace (black). The time axis is linear until 15 ps and logarithmic thereafter.

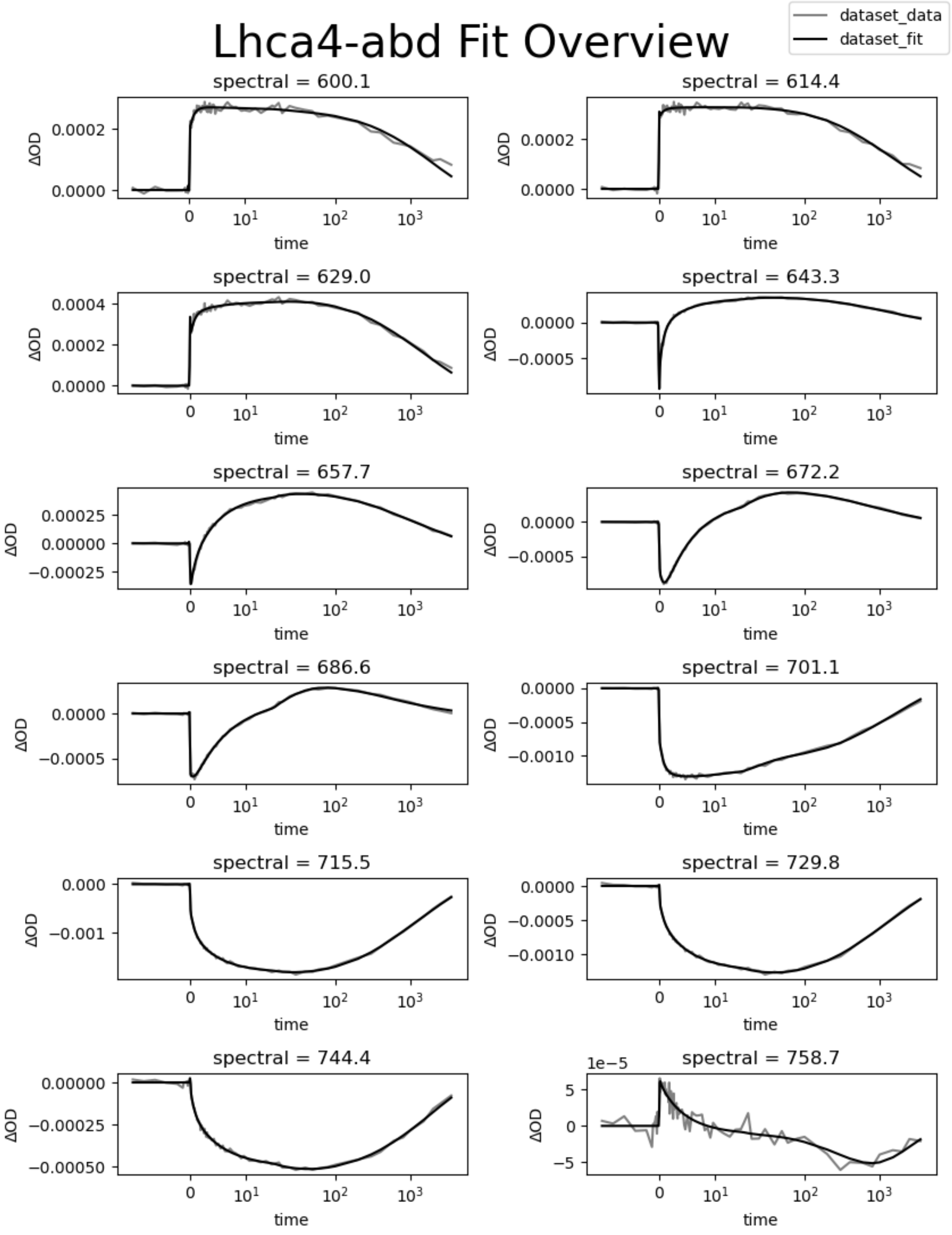

Figure S7. Fitting quality of the global analysis of the Lhca4-abd sample. For a selection of wavelengths the time-trace of the raw data of the transient absorption experiment (grey) is shown along the globally fitted trace (black). The time axis is linear until 15 ps and logarithmic thereafter.

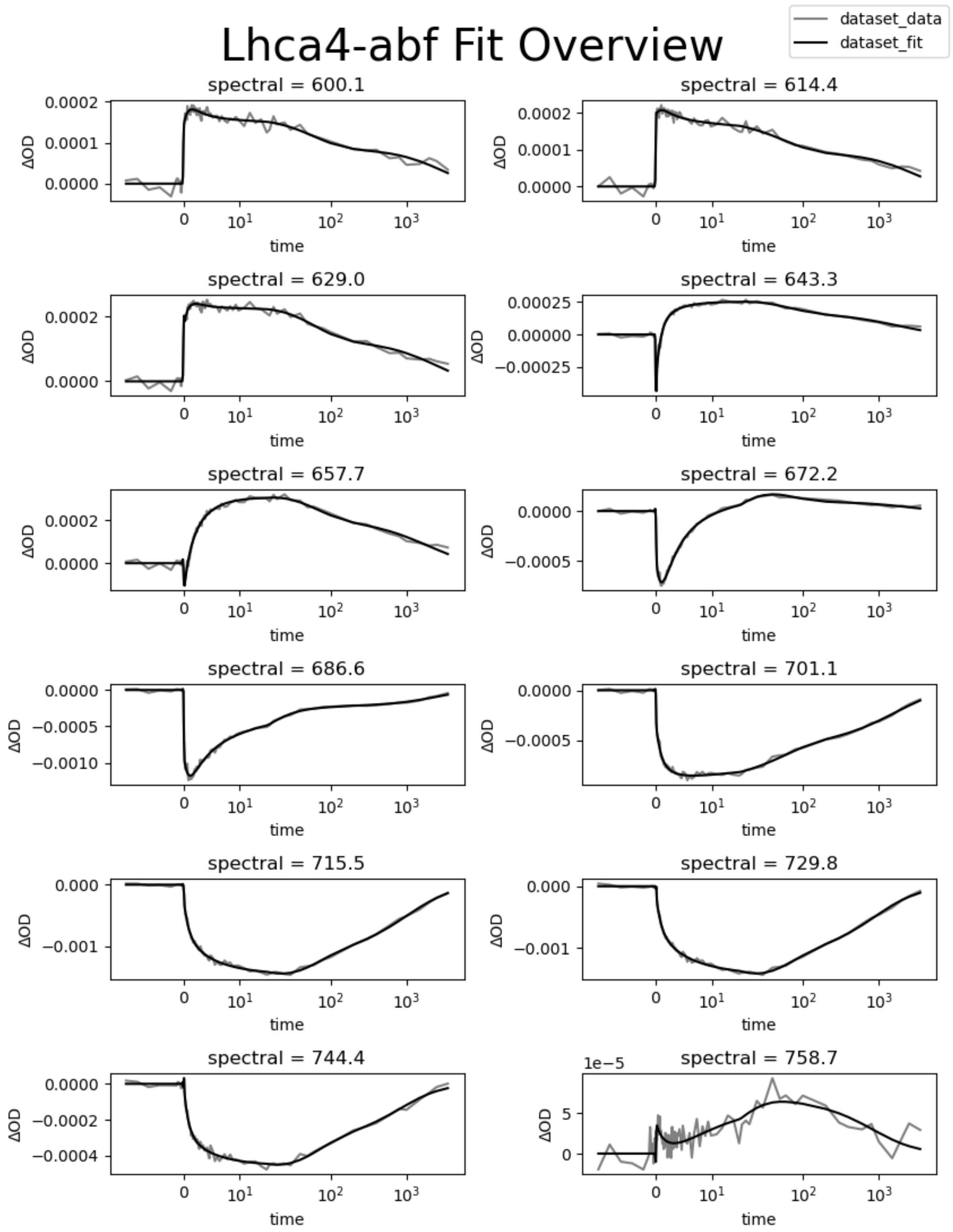

Figure S8. Fitting quality of the global analysis of the Lhca4-abf sample. For a selection of wavelengths the time-trace of the raw data of the transient absorption experiment (grey) is shown along the globally fitted trace (black). The time axis is linear until 15 ps and logarithmic thereafter.

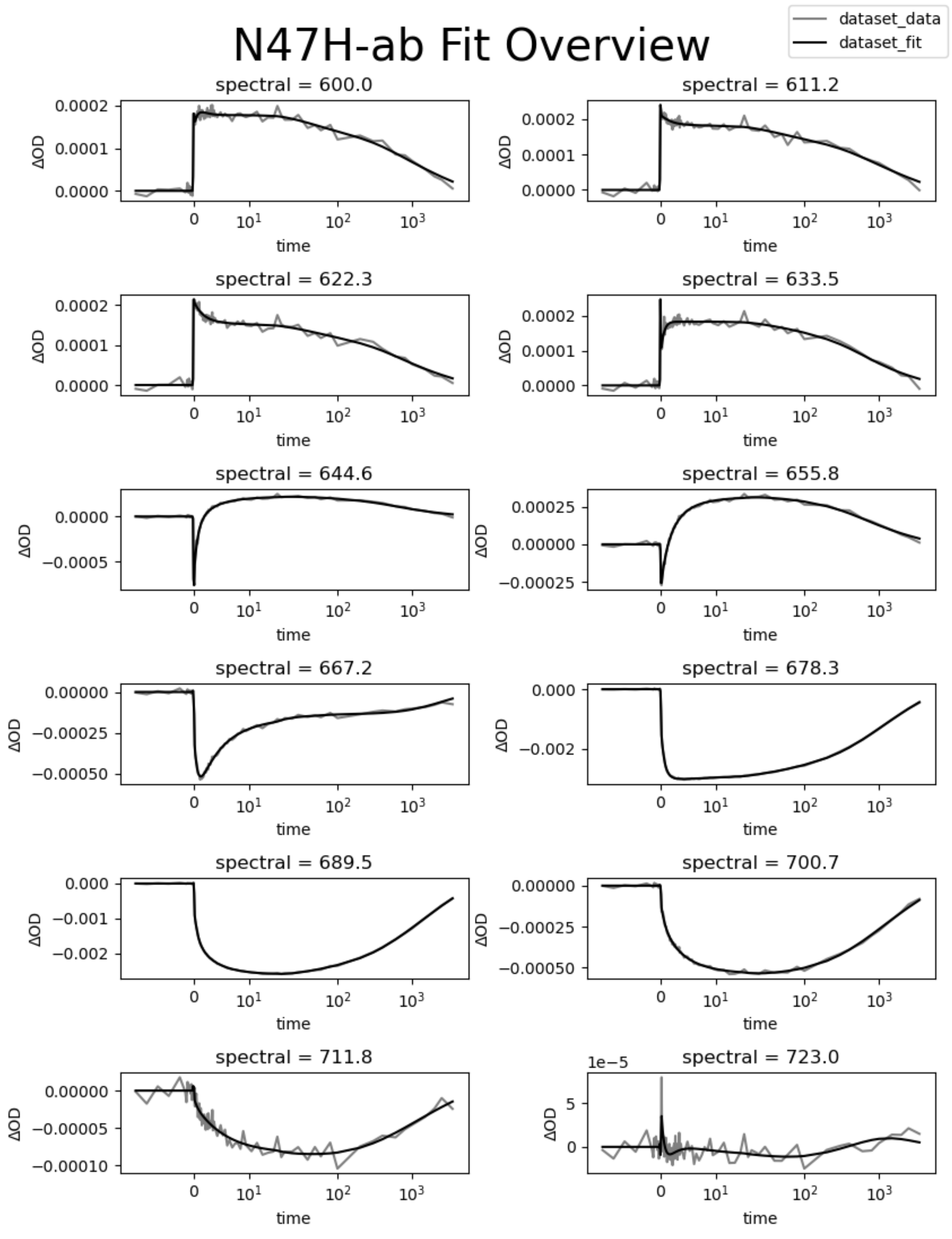

Figure S9. Fitting of the N47H-ab sample. For a selection of wavelengths the time-trace of the raw data of the transient absorption experiment (grey) is shown along the globally fitted trace (black). The time axis is linear until 15 ps and logarithmic thereafter.

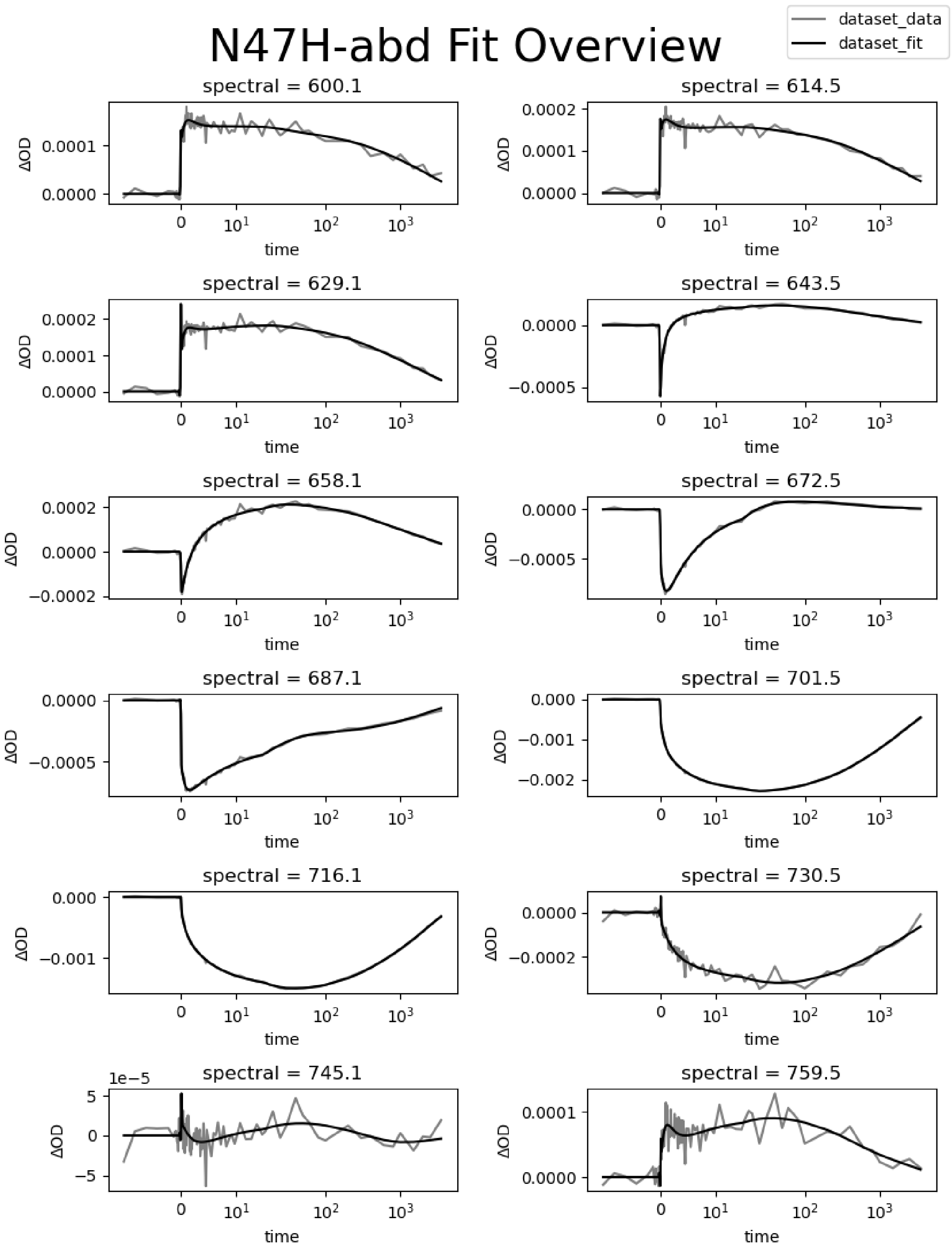

Figure S10. Fitting of the N47H-abd sample. The time-trace of the raw data of the transient absorption experiment for selected wavelength (grey) is shown along with the globally fitted trace (black). The time axis is linear until 15 ps and logarithmic thereafter.

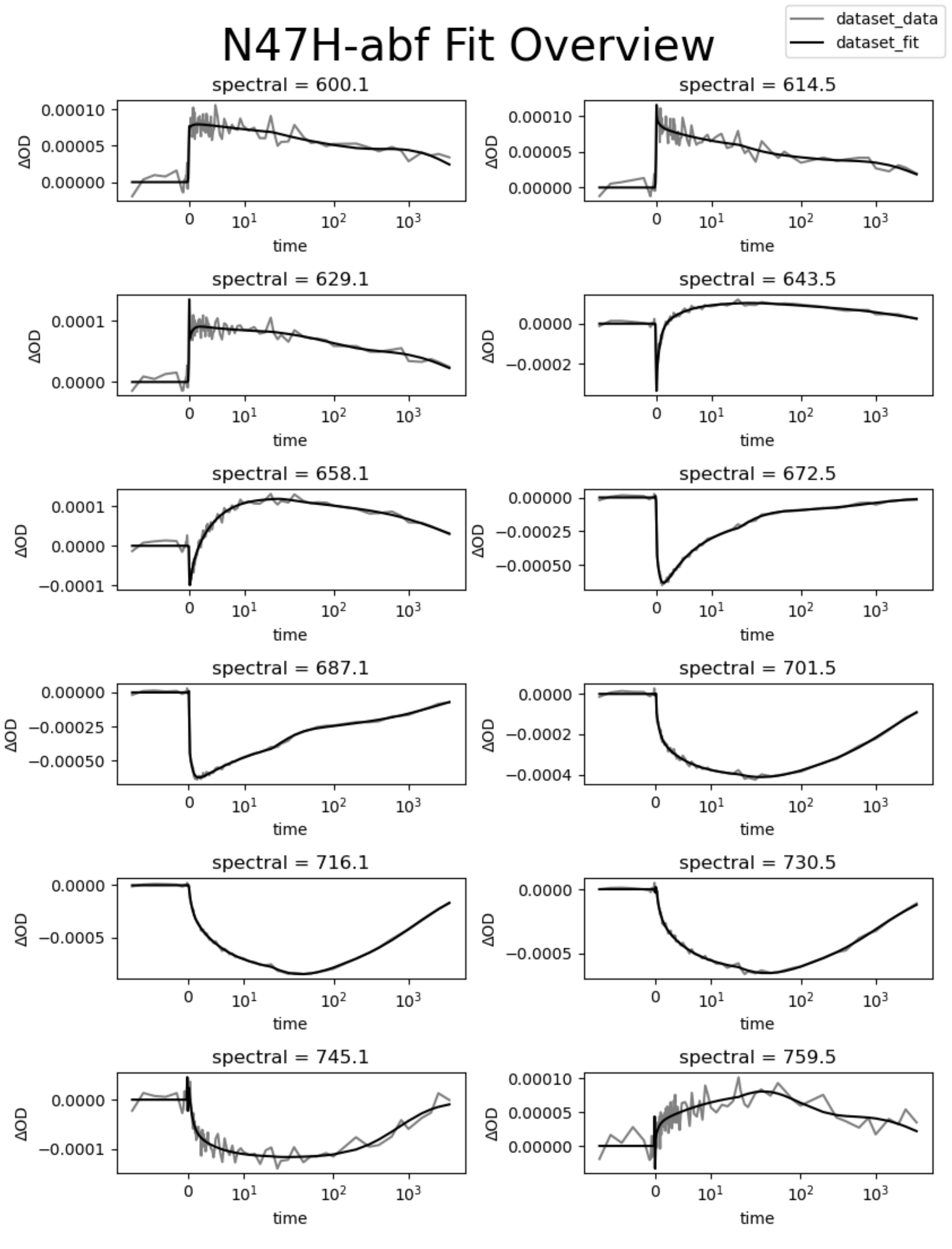

Figure S11. Fitting of the N47H-abf sample. The time-trace of the raw data of the transient absorption experiment for selected wavelengths (grey) is shown along with the globally fitted trace (black). The time axis is linear until 15 ps and logarithmic thereafter.

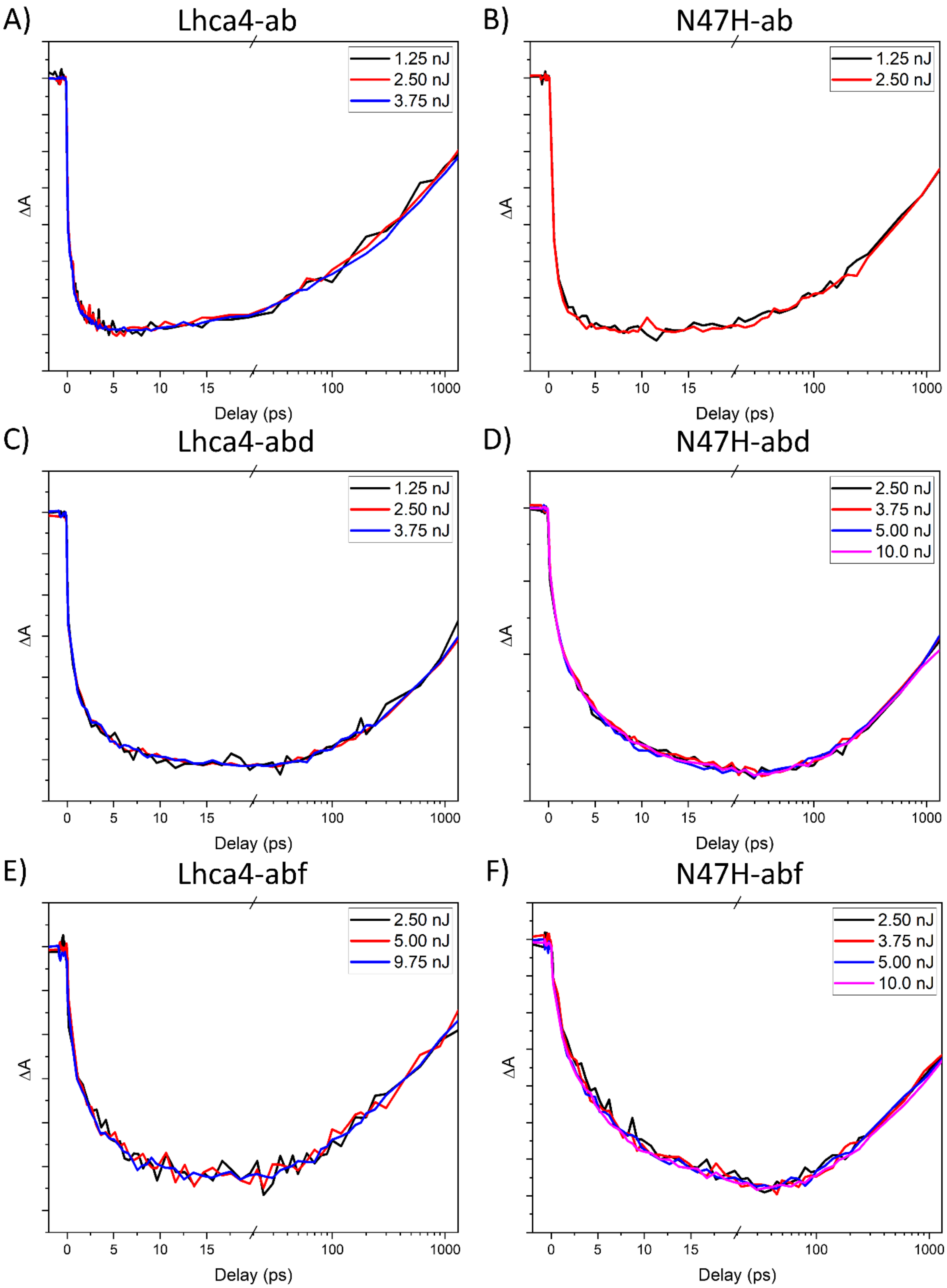

Figure S12. Power studies for the transient absorption measurements of the Lhca4 and N47H samples excited at 642 nm. Time-traces at several pulse energies extracted at the wavelength where the amplitude in the most strongly quenched DADS (43.4 ps, 36.0 ps, 24.0 ps, 19.1 ps, 53.5 ps and 91.1 ps respectively) is the largest. Lhca4-ab **(A)**, N47H-ab **(B)**, Lhca4-abd **(C)**, N47H-abd **(D)**, Lhca4-abf **(E)** and N47H-abd **(F)**.

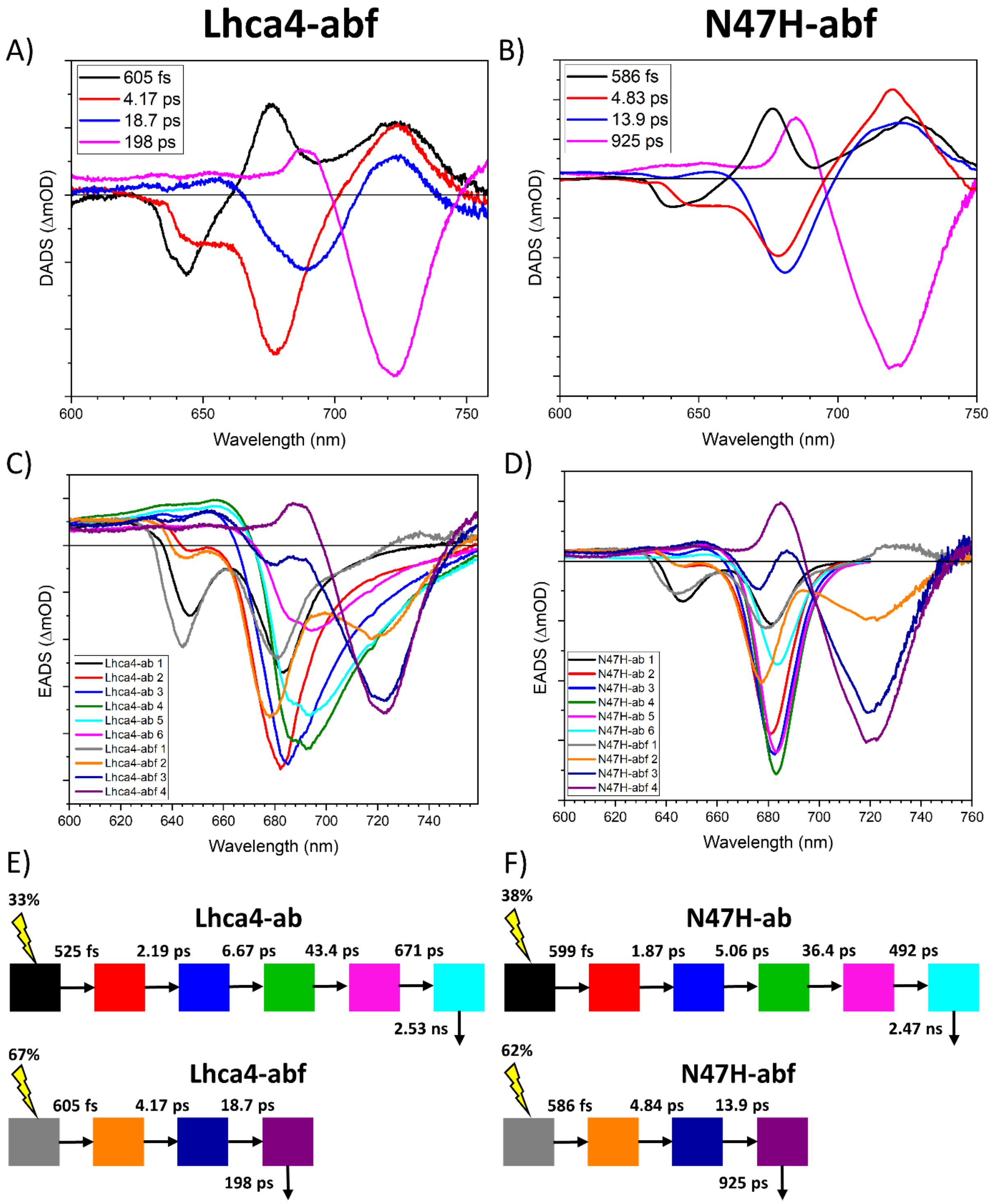

Figure S13. Target analysis results for the samples reconstituted with Chl *f*. DADS for the independent sequential scheme that represents the complexes with Chl *f* in the Lhca4-abf sample **(A)** and in the N47H-abf sample **(B)**. For better visibility the N47H-abf DADS in **(B)** have been smoothed following a Savitzky-Golay algorithm in the region >720 nm, while paying attention to preserve the original shape of the DADS. The full set of EADS for the target analyses for the Lhca4-abf **(C)** and N47H-abf **(D)** sample are also shown. The kinetic schemes that correspond to these EADS for the Lhca4-abf sample **(E)** and N47H-abf **(F)** are represented below. The lightning bolts above each of the sequential branches in the kinetic schemes represent the initial excitation vector. The colour code used for the kinetic schemes in **E** & **F** is the same for the set of EADS in **C** & **D**.

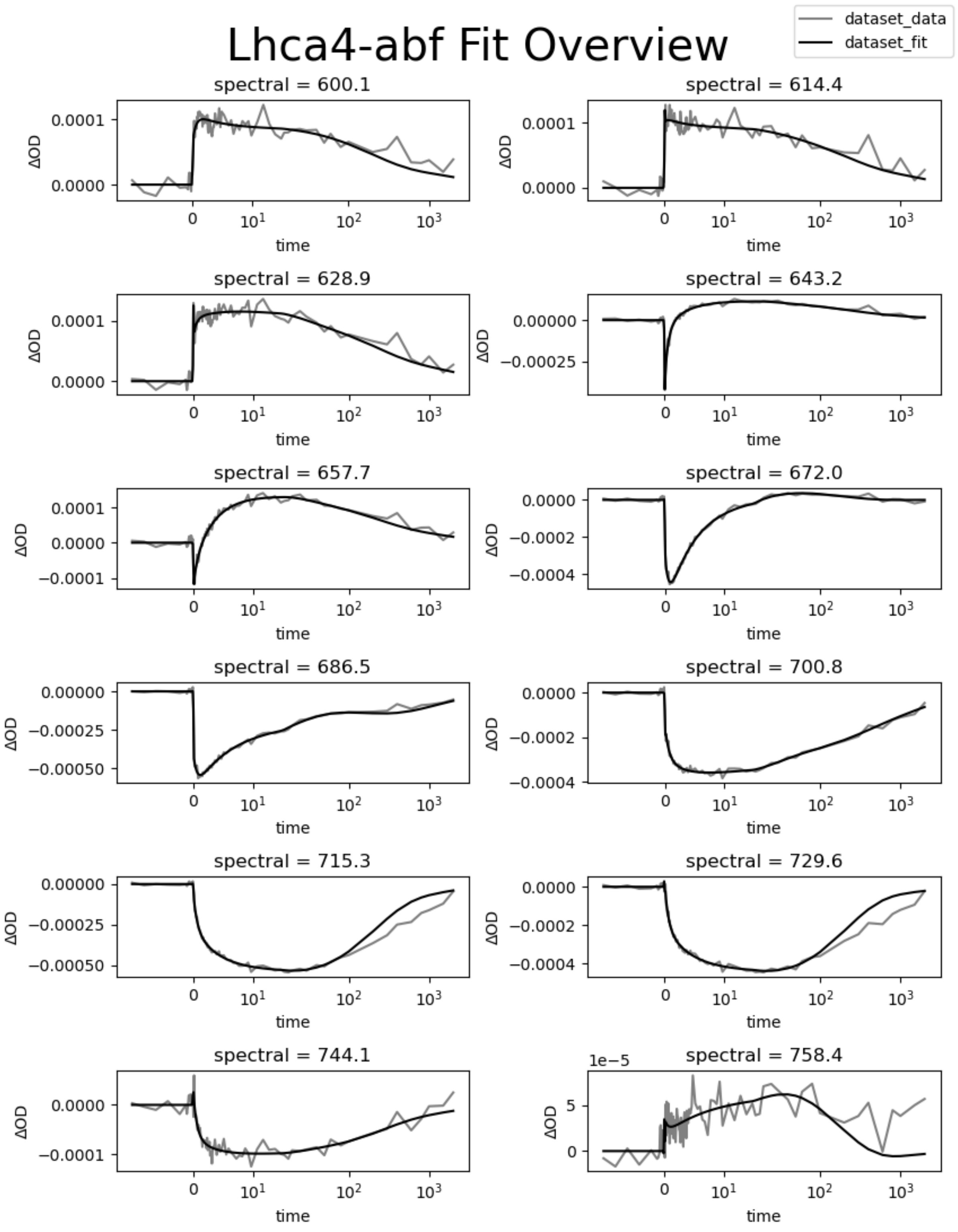

Figure S14. Target fittings of the Lhca4-abf sample. The time-trace of the raw data of the transient absorption experiment for selected wavelengths (grey) is shown along with the globally fitted trace (black). The time axis is linear until 15 ps and logarithmic thereafter. Note that the fit quality after 100 ps is poor as this was outside of the fitted time-window.

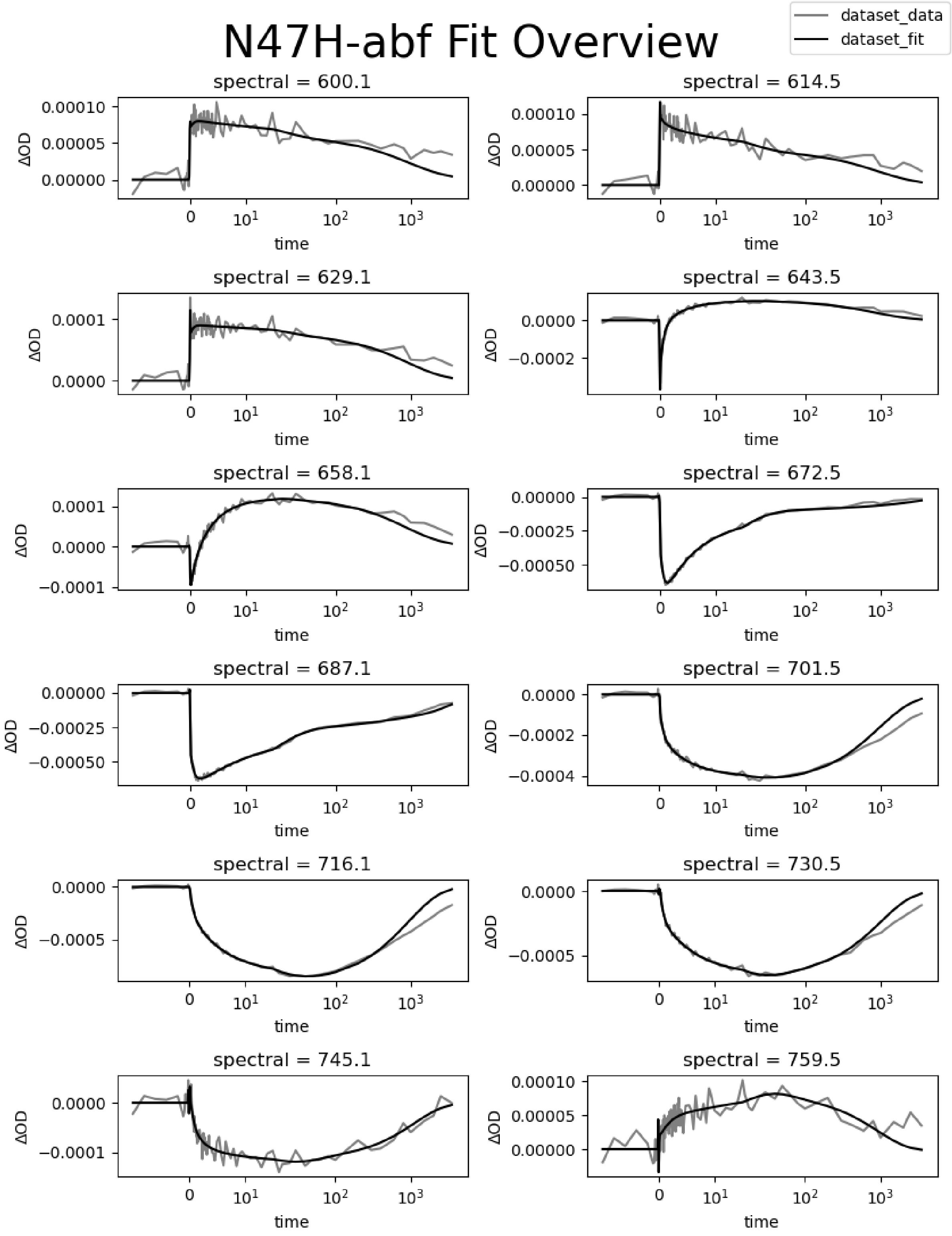

Figure S15. Target fittings of the N47H-abf sample. The time-trace of the raw data of the transient absorption experiment for selected wavelengths (grey) is shown along with the globally fitted trace (black). The time axis is linear until 15 ps and logarithmic thereafter. Note that the fit quality after 100 ps is poor as this was outside of the fitted time-window.

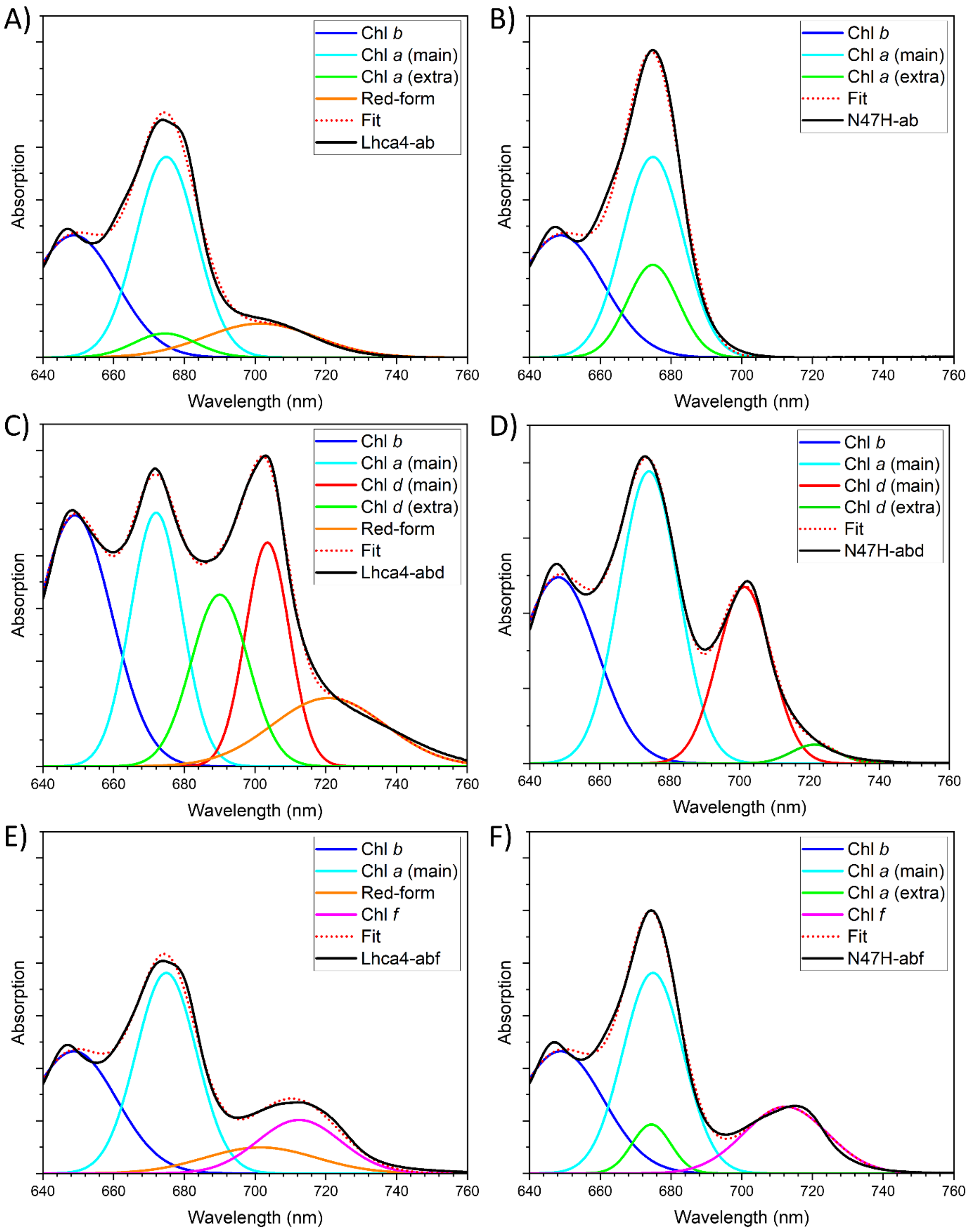

Figure S16. Gaussian deconvolution of 77 K absorption spectra of the *ab*, *abd* and *abf* containing complexes. The fitted Gaussians (colored lines) are shown together with their sum (red dotted lines) and the measured absorption spectra (black line) for Lhca4-ab (**A**), N47H-ab (**B**), Lhca4-abd (**C**), N47H-abd (**D**), Lhca4-abf (**E**) and N47H-abf (**F**).
